# Mast Cell Infiltration in Human Brain Metastases Modulates the Microenvironment and Contributes to the Metastatic Potential

**DOI:** 10.3389/fonc.2017.00115

**Published:** 2017-06-02

**Authors:** Ananya Roy, Sylwia Libard, Holger Weishaupt, Ida Gustavsson, Lene Uhrbom, Göran Hesselager, Fredrik J. Swartling, Fredrik Pontén, Irina Alafuzoff, Elena Tchougounova

**Affiliations:** ^1^Department of Immunology, Genetics and Pathology, Uppsala University, Rudbeck Laboratory, Uppsala, Sweden; ^2^Department of Biomedical Sciences and Veterinary Public Health, Swedish University of Agricultural Sciences, Uppsala, Sweden; ^3^Department of Neurosurgery, Uppsala University, University Hospital, Uppsala, Sweden; ^4^Science for Life Laboratory, Uppsala University, Uppsala, Sweden

**Keywords:** mast cell, brain metastases, IL-8, IL-10, matrix metalloprotease 2, vascular endothelial growth factor

## Abstract

Metastatic brain tumors continue to be a clinical problem, despite new therapeutic advances in cancer treatment. Brain metastases (BMs) are among the most common mass lesions in the brain that are resistant to chemotherapies, have a very poor prognosis, and currently lack any efficient diagnostic tests. Predictions estimate that about 40% of lung and breast cancer patients will develop BM. Despite this, very little is known about the immunological and genetic aberrations that drive tumorigenesis in BM. In this study, we demonstrate the infiltration of mast cells (MCs) in a large cohort of human BM samples with different tissues of origin for primary cancer. We applied patient-derived BM cell models to the study of BM cell–MC interactions. BM cells when cocultured with MCs demonstrate enhanced growth and self-renewal capacity. Gene set enrichment analyses indicate increased expression of signal transduction and transmembrane proteins related genes in the cocultured BM cells. MCs exert their effect by release of mediators such as IL-8, IL-10, matrix metalloprotease 2, and vascular endothelial growth factor, thereby permitting metastasis. In conclusion, we provide evidence for a role of MCs in BM. Our findings indicate MCs’ capability of modulating gene expression in BM cells and suggest that MCs can serve as a new target for drug development against metastases in the brain.

## Introduction

Metastasis to the brain is a major reason of high mortality in patients with systemic cancers. Metastatic brain tumors occur in about 25% of all cancer patients and have a high mortality rate. The median survival of patients diagnosed with brain metastases (BMs) and treated with aggressive therapies is generally 4–12 months (1). The annual incidence of BM has been increasing the past decade and population-based studies predict 10–14 new BM cases per 100,000 persons (2). This increase in BM is speculated to be a consequence of the relatively prolonged patient life, which is a result of improved primary cancer treatment and imaging techniques (3, 4).

Brain metastasis differs in many aspects from metastases in other organs and is a treatment challenge owing to the progressive neurological disability and the lack of any effective treatment due to the unique structure of the blood–brain barrier (BBB). Even though the brain has been considered as an immune-privileged organ and not much is known about the inflammatory response by circulating tumor cells, BMs do contain infiltrating immune cells (5–7). The presence of tumor-infiltrating lymphocytes (TILs) is associated with favorable patient survival, whereas microglia/macrophage infiltration seems to provide a premetastatic niche for the BM initiating cells and promotes growth and survival of tumor cells in the central nervous system (CNS) microenvironment (8). Finally, the available data from recent studies show that BM harbors an active inflammatory microenvironment (5, 6, 9, 10), which may be exploited as a treatment target.

Mast cells (MCs) are versatile immune cells that have been implicated in various pathophysiological conditions including cancer (11, 12). They are usually dispersed throughout most tissues in their mature form or circulate in the blood as progenitors prepared to infiltrate the tissue on demand (13). MCs produce and store a variety of mediators and are armed with diverse receptors. Thus, they are sensitive to environmental modifications and, upon stimulation, they are able to secrete several biologically active factors involved in the modulation of tumor growth. MCs are long-lived secretory cells whose activity, magnitude, and nature of response to stimulation are regulated and fine-tuned by environmental as well as genetic factors. Hence, even though MCs are not the most abundant of immune cells, given the ability to instantly release various mediators from their intracellular stores (14), shape immune responses (15), angiogenesis (16), tumor development (17), and tissue remodeling (11); they are crucial for optimal immune responses during inflammation and tumorigenesis. The overall impact of MCs in the tumor microenvironment needs however detailed investigation since the prognostic significance of MC infiltration in solid tumors seems to be highly dependent on the type and stage of cancer (18).

In this study, we report that MCs infiltrate human BM and in line with this finding we provide evidence for a role of MCs in BM. We show that MCs *via* IL-8, IL-10, vascular endothelial growth factor (VEGF), and matrix metalloprotease 2 (MMP2) can modulate the BM tissue microenvironment and thereby induce growth and propagation of the BM cells. We also identify a set of candidate genes that are overexpressed in BM cells upon coculture with MCs and demonstrate that MCs can support and boost the self-renewal capacity of the BM cells. Taken together, our results show the presence of MCs in BM and indicate that MCs provide a microenvironment favorable for the development and progression of BM.

## Materials and Methods

### Clinical Samples

Permission for use of human tissue samples for this study was obtained from the Ethics Committee of Uppsala, Sweden (Dnr 2014/535). The study involving human tissue samples was conducted in accordance with the Declaration of Helsinki and the patients gave written informed consent for the sample collection. All human tissue samples and related patient records for research purpose (as listed in Table S1 in Supplementary Material) are part of Uppsala Biobank material and were provided to the researchers as per ethical permission and all material obtained in compliance with the Declaration of Helsinki. The researchers did not have any interaction with any patients and were not involved in the collection of human patient samples during the course of this study. Patient identity was anonymous for the researchers. All human tumor tissue sections thereafter were evaluated based on the WHO classification by experienced neuropathologists.

### Cell Cultures

All cells were cultured at 37°C under 5% CO_2_. U3333MET, a human BM cell line was cultured in 10% FBS-containing MEM supplemented with 4 mM l-glutamine, 100 U/ml penicillin, and 0.1 mg/ml streptomycin. The U3333MET cell line was established in our lab after surgery from a patient with BM. The patient had been earlier diagnosed with primary lung cancer. NCI-H1915 cell was obtained from ATCC and was cultured in 10% FBS-containing modified RPMI-1640 supplemented with 4 mM l-glutamine, 100 U/ml penicillin, and 0.1 mg/ml streptomycin. NCI-H1915 is a BM cell line from a patient with lung cancer.

The human MC line LAD2 (obtained from Prof Dean Metcalfe at NIH/NIAID, MD, USA) was cultured as described previously (19) in StemPro medium supplemented with 4 mM l-glutamine, 100 U/ml penicillin, and 0.1 mg/ml streptomycin and 100 ng/ml SCF (300-07, Peprotech).

### Coculture Assays

To examine the effect of MCs on BM cell growth and secretion, LAD2 cells were cocultured in 6-well format transwell (0.4 µm) with the two BM cell lines for 12, 24, and 48 h. Briefly, the BM cell lines were plated on 6-well plates in low serum (1%) conditions and allowed to attach for 2–3 hours. Overnight SCF starved LAD2 cells were suspended in medium (5 × 10^5^ cells/ml) and added to the transwell. The cocultures are left to grow undisturbed for 12, 24, and 48 h. Stimulation experiment was done in triplicates. Appropriate negative controls were kept for each experiment.

### β-Hexosaminidase Release Assay

To measure the level of MC degranulation induced by the BM cells, LAD2 cells (1 × 10^6^ cells/ml) in triplicates were incubated at 37°C in 5% CO_2_ in Hanks balanced salt solution for 1 h in the presence of either 2 µM calcium ionophore A23187 (as a positive control) or for 4 h in coculture with BM cells. Samples were taken at each time point and cells were centrifuged at 300 *g* for 10 min. Supernatants were incubated with 1 mM *p*-nitro-phenyl-*N*-acetyl-β-d-glucosaminide (N9376, Sigma Aldrich) in 0.05 M citrate buffer (pH 4.5) at 37°C for 1 h. As a control for total β-hexosaminidase content, cells were lysed with 1% Triton X-100 and incubated as above. All reactions were quenched by addition of 100 µl of 0.05 M Na_2_CO_3_ (pH 10.0) and the absorbance was read at 405 nm.

### AlamarBlue Assay

Brain metastasis cells were seeded in triplicates and allowed to attach overnight. The following day medium was removed and replaced with either control medium (medium supplemented with 1% FBS) or 0.4 µm transwell with LAD2 cells. The cells were then grown for 12, 24, and 48 h. Cell proliferation was assayed by adding AlamarBlue (88951, ThermoFischer) according to the manufacturer’s recommendations.

### EdU Proliferation Assay

Cell proliferation was assayed with the Click-iT^®^ Plus EdU Alexa Fluor^®^ 555 imaging kit following manufacture’s recommendation (C10638, Life Technologies). The assay is based on the principle of efficiently incorporating modified thymidine analog EdU (5-ethynyl-2′-deoxyuridine, a nucleoside analog of thymidine) into newly synthesized DNA. Visualization is achieved by fluorescent labeling with a bright, photostable Alexa Fluor^®^ dye in a fast, highly specific, mild click reaction. Briefly, BM cells were seeded on cover slips and allowed to attach overnight. Following this, they were grown alone in normal media or in coculture with MCs for a period of 48 h. EdU was added 4–5 h prior to the end point of experiment. Cells were fixed, permeabilized, and EdU positive cells were detected with Alexa Fluor^®^ 555-conjugated secondary antibodies and cell nuclei were detected by Hoechst 33342. This was followed by image acquisition and analysis.

### Wound Healing Assay

Confluent monolayers of U3333MET and NCI-H1915 cells were scratched by a razor blade from more than four replicate plates for each cell type. The cells were then left in the incubator with either normal growth medium or transwell inserts with LAD2 cells on top. Images of the similar areas of scratches were taken immediately after scratching, 24 h and 48 h post-scratching by Nikon Eclipse TS 100 microscope. Quantification of wound closure was done by using the ImageJ MRI Wound Healing Tool. It measures the area of a wound in a cellular tissue on a stack of images representing a time series. Data are presented as percentage of wound closure.

### Tumorsphere Formation Assay

To evaluate the cancer stem/progenitor cell property of the BM cells before and after MC coculture, the *in vitro* tumorsphere formation assay was performed as described elsewhere (20). Briefly, confluent monolayer of NCI-H1915 or U3333MET cells with or without 48 h coculture with LAD2 cells were detached by trypsinization, and centrifuged. The resulting pellet was then suspended in 5 ml of 1× PBS. The cells were counted and the dilution was adjusted with the appropriate volume of tumorsphere medium to make the cell concentration at 1 cell/l. The cells were kept on ice while not in use for the entire duration of the experiment. A total of 200 µl of the cells suspension in tumorsphere medium was then seeded into each well (200 cells per well) of 96-well ultra-low attachment round bottom plates. For each cell line or treatment, 2 rows for a total of 20 wells were used such that a total of 4,000 cells per treatment were seeded. The upper and lower edges of the 96-well plate was sealed with tape to avoid evaporation of medium and the plate was left undisturbed in 37°C, with 5% CO_2_ for a week. After 7–8 days, tumorsphere numbers (sized between 50 and 200 µm) were counted under Nikon Eclipse TS 100 microscope. Data are presented as percentage of the number of tumorspheres divided by the initial number of cells seeded (4,000 cells).

### Cytokine Array and ELISA

The human cytokine array kit (ARY005, R&D Systems) was used according to the manufacturer’s instructions. In brief, LAD2 cells were grown alone or in coculture with BM cells. Supernatant from the cultures were collected and applied to membranes overnight, after which signals were detected after appropriate application of antibody cocktails and streptavidin–HRP solutions. Quantification of the duplicate spots on the filters was done using ImageJ software as instructed by the manufacturers.

SCF starved LAD2 cells were grown alone or in coculture with BM cells. IL-8 and IL-10 levels in culture supernatants were measured using a quantitative immunoassay ELISA kit (900K21, 900K18, Peprotech) following manufacturer’s protocol. Both ELISA were done in triplicates for each cell experiment.

### RNA Extraction, cDNA Synthesis, and qPCR

RNA was extracted from control LAD2 and BM cells as well as from LAD2 and BM cells after coculture. RNA extraction was done using GENEJET RNA Purification Kit (Life Technologies) extraction method from cell pellets. cDNA was synthesized using the High-Capacity RNA-to-cDNA™ Kit (4387406, Thermo Fisher Scientific), which was then used to perform the qPCR using the PowerUp SYBR Mastermix. The target-specific primers used are listed in Table S2 in Supplementary Material. For all qPCR analysis, β-actin expression was used as endogenous control. Results are presented as fold induction. The experiments were performed three times, with triplicates in each case.

### Microarray Expression Analysis

RNA quality was evaluated using the Agilent 2100 Bioanalyzer system (Agilent Technologies Inc., Palo Alto, CA, USA). In all, 100 ng of total RNA from each sample was used to generate amplified and biotinylated sense-strand cDNA from the entire expressed genome according to the GeneChip^®^ WT PLUS Reagent Kit User Manual (P/N 703174 Rev. 1, Affymetrix Inc., Santa Clara, CA, USA). GeneChip^®^ ST Arrays (GeneChip^®^ Human Gene 2.0 ST Array) were hybridized for 16 h in a 45°C incubator, rotated at 60 rpm. According to the GeneChip^®^ Expression Wash, Stain and Scan Manual (P/N 702731 Rev. 3, Affymetrix Inc., Santa Clara, CA, USA), the arrays were then washed and stained using the Fluidics Station 450 and finally scanned using the GeneChip^®^ Scanner 3000 7G. The analysis was performed at the Array and Analysis Facility, Science for Life Laboratory at Uppsala Biomedical Centre, Uppsala, Sweden.

### Microarray Data Analysis

The raw data were normalized in Expression Console, provided by Affymetrix (http://www.affymetrix.com), using the robust multi-array average method that was first suggested by Li and Wong in 2001 (21, 22).

### Immunohistochemistry and Immunofluorescence

Formalin-fixed, paraffin-embedded 6 µm thick tissue sections were fixed. Thereafter, the sections were deparaffinized (in xylene on a rocking table for 1 h × 2 h followed by 2 min × 5 min incubations in 100% EtOH, 95% EtOH, 80% EtOH, distilled H_2_O) and subjected to pressure boiling for antigen retrieval in antigen unmasking solution (Vector Labs). Immunohistochemistry was performed using the UltraVision LP detection System (Thermo Fisher Scientific) in accordance with the manufacturer’s instructions. Briefly, after antigen retrieval, the slides were washed in PBS-T [containing 0.05% Tween (Sigma Aldrich)] and incubated with hydrogen peroxidase block. Ultra V block was subsequently applied. Primary antibody used was anti-human tryptase (sc-33676, Santa Cruz Biotechnology) diluted in 5% milk in PBS-T. Primary antibody was applied overnight at 4°C, followed by primary antibody enhancer. Slides were incubated with HRP polymer, and the signal was visualized using freshly prepared DAB plus chromogen and substrate mix. Between all the steps described above, the slides were thoroughly washed in PBS-T. After the final step, the slides were washed in distilled H_2_O, counterstained with hematoxylin and mounted using Immu-mount (Thermo Fisher Scientific).

For immunofluorescence staining, coverslips were rinsed in PBS, blocked in 5% milk-containing PBS-T [supplemented with 0.2% Triton X-100 (Sigma Aldrich)] for 1 h, followed by overnight incubation (4°C) with the primary antibody diluted in the blocking solution. The coverslips were subsequently incubated with appropriate secondary antibody for 45 min. Nuclei were stained with DAPI (1:5,000) for 15 min and mounted in Immu-mount. All secondary antibodies used were Alexa antibodies (Invitrogen).

### Image Analysis

IHC and IF stained slides were imaged using ZEISS AxioImager for brightfield (AxioCam color) and fluorescence (AxioCam monochrom) and Zen Blue software. Image analysis was done using ImageJ software.

### In-Cell ELISA

In-cell ELISA (62200, Invitrogen) was used to determine the relative protein levels in whole BM cells before and after coculture with MCs according to the manufacturer’s protocol with slight modification. It is an accurate and efficient method of analysis of protein levels in cells and is ideal since it can be performed on a 96-well format with multiple repeats and less cell number. Briefly, the BM cells were seeded and allowed to attach for 3–4 h following which they were grown subsequently alone or in coculture with LAD2 cells. All experiments were performed under reduced serum condition. After the stipulated time period, the wells were washed with ice cold PBS and then proceeded according to the kit protocol. Human SOX2 (AB5603, Merck Millipore) and human CD133 (Ab19898, Abcam) antibodies were used and the detected with a horseradish peroxidase conjugate. Cell number normalization was done with the whole-cell stain, Janus Green. Absorbance was detected using an ELISA plate reader.

### Bioinformatics Analysis

In order to assess the differentially expressed genes (DEGs) in the normal and LAD2 cocultured NCI-H1915 and U3333MET cells, the enrichment of functional/pathway annotations was investigated through the bioinformatic resource Database for Annotation, Visualization and Integrated Discovery (DAVID). Gene set enrichment analysis (GSEA) and pathway analysis was performed on the Broad Institute- MSigDB (The Molecular Signatures Database). To address the problem with multiple testing, the *p*-values were adjusted using the method by Hochberg and Benjamini (23). The heat map and clustering was done using the Genesis software (24).

### Statistical Analysis

Statistical analyses were done using the GraphPad Prism software (GraphPad Software 6.0d). For groupwise comparisons, the Student’s unpaired *t*-test was used. For comparisons between more than two groups, ANOVA was applied.

## Results

### MCs Infiltrate Human BMs

**Figure 1:**
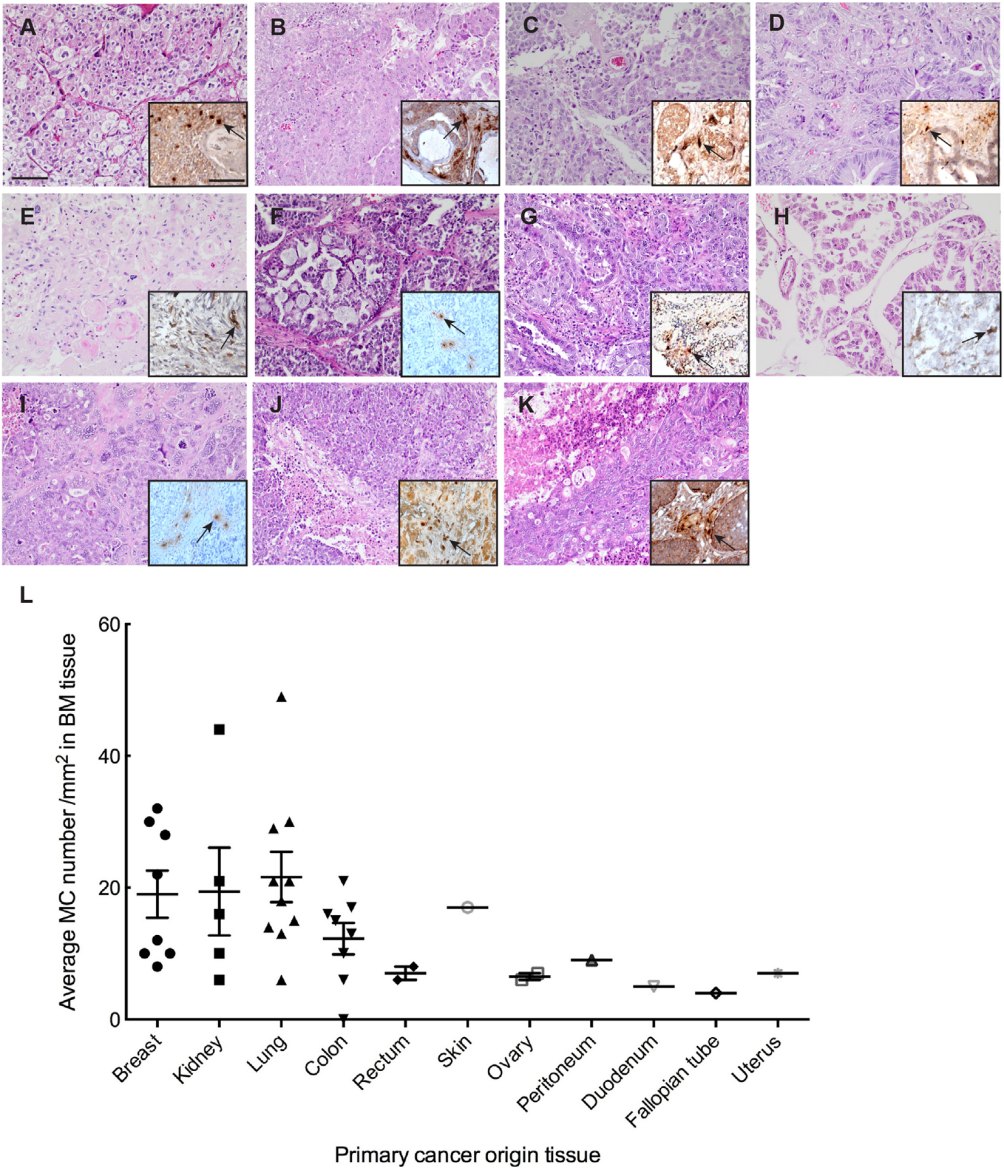
Mast cell (MC) infiltration in brain metastasis (BM). **(A–K)** Representative pictures of H&E staining of BM with different primary cancer origin. **(A)** kidney, **(B)** colon, **(C)** breast, **(D)** rectum, **(E)** skin, **(F)** ovary, **(G)** lung, **(H)** peritoneum, **(I)** fallopian tube, **(J)** uterus, **(K)** duodenum. Inserts to the right in each picture: representative picture of IHC staining for MC tryptase showing MC infiltration in the BM tissue with different primary cancer origin. Pictures are taken at 20× magnification. **(L)** Quantification of MC infiltration in BM of patients from different primary tumor origin.

To study the extent of MC infiltration in BMs, we analyzed tryptase (TPSAB1) expression profiles in BM tissues from 40 patients (Table S1 in Supplementary Material). We examined 31 metastatic adenocarcinoma nodules, 8 metastatic renal carcinoma nodules, and 1 metastatic squamous carcinoma nodule of diverse origin (lung, breast, prostate, colorectal, uterus, ovary) with hematoxylin eosin staining followed by evaluation for metastases formation and primary cancer origin (Figure 1). These sections were subsequently screened for MC infiltration by staining for tryptase (Figure 1). We were able to detect MC infiltration in almost all BM tissues and the MC numbers were significantly higher in BM tissues with lung, breast, and kidney as primary cancer origin. MC distribution pattern in the tissues varied, and they were localized mostly in clusters but in scattered individual groups as well. In samples containing both normal parenchyma as well as clear tumor regions MCs were observed in vicinity to vessels and were significantly localized adjacent to tumor cells or in areas where the majority of cells were tumor cells.

### BM Cells Induce MC Activation and Migration

**Figure 2:**
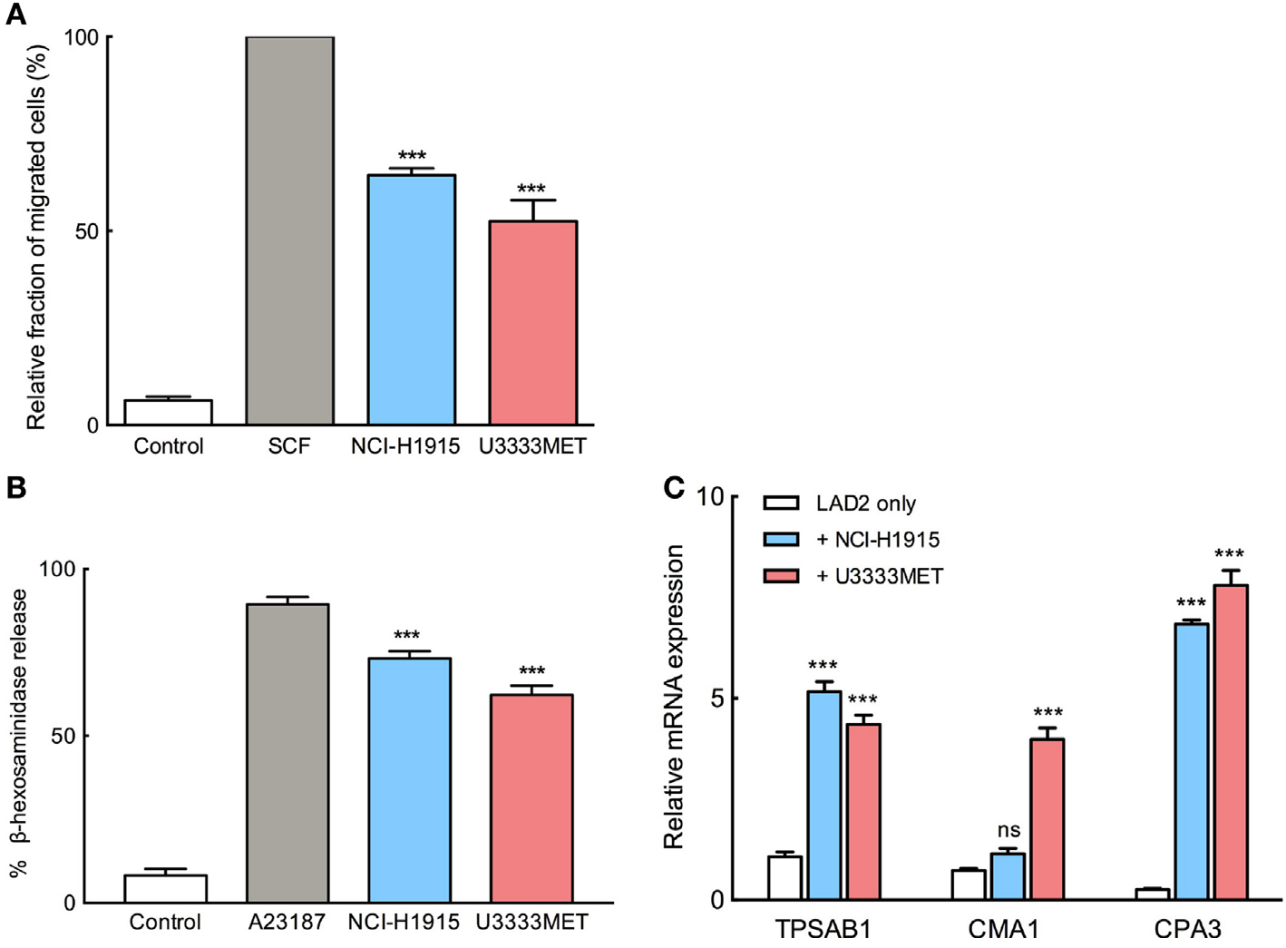
Migration and activation of mast cells (MCs) in response to brain metastasis (BM) cells. **(A)** Migration of MCs toward NCI-H1915 or U3333MET cells measured by transwell assay. Medium without serum was used as negative and SCF as positive chemotactic control. **(B)** β-hexosaminidase release by MCs in response to stimulation by NCI-H1915 or U3333MET cell lines as indicated. 2 µM calcium ionophore A23187 was used as a positive control and HBSS as negative control. **(C)** Quantitative PCR to evaluate mRNA levels of the MC-specific proteases in response to the indicated BM cell line. Unstimulated LAD2 cells were used as control. β-actin mRNA detection was used for normalization. The experiments were performed three times in triplicates and mean values + SEM was plotted, ns, not significant; **p < 0.01, ***p < 0.001.

To elucidate the possible mechanisms underlying the accumulation of MCs in BM, a number of experiments were performed. We used the two cell lines, NCI-H1915 and U3333MET, developed from BMs with primary lung carcinoma origin. To evaluate the recruitment possibility, we performed migration assay, in which SCF starved LAD2 cells were placed into hanging inserts and were allowed to actively migrate through an 8 µm porous membrane toward BM cells. MC migration toward BM cells was almost 60% as effective as migration toward SCF (Figure 2A). We conclude that BM cells can recruit MCs by secreting various chemoattractants. As a next step, we wanted to evaluate whether MC chemotaxis is accompanied by activation of MCs. The starved LAD2 cells were stimulated with U3333MET or NCI-H1915 cells and the supernatants were then subjected to β-hexosaminidase release assay. A significant increase in β-hexosaminidase as compared to unstimulated control was observed (Figure 2B). Along with this, the expression of the MC-specific proteases, tryptase (TPSAB1), chymase (CMA1), and carboxypeptidase A3 (CPA3) was also assessed. A consistent and significant increase in expression of all the three proteases was observed in LAD2 cells when cocultured with U3333MET cells. For the LAD2 cells cocultured with NCI-H1915 cells, only the expression increase of tryptase and CPA3 was significant (Figure 2C). Taken together, these results indicate that BM cells can induce MC degranulation followed by activation.

### MCs Support the Growth and Propagation of BM Cells

**Figure 3:**
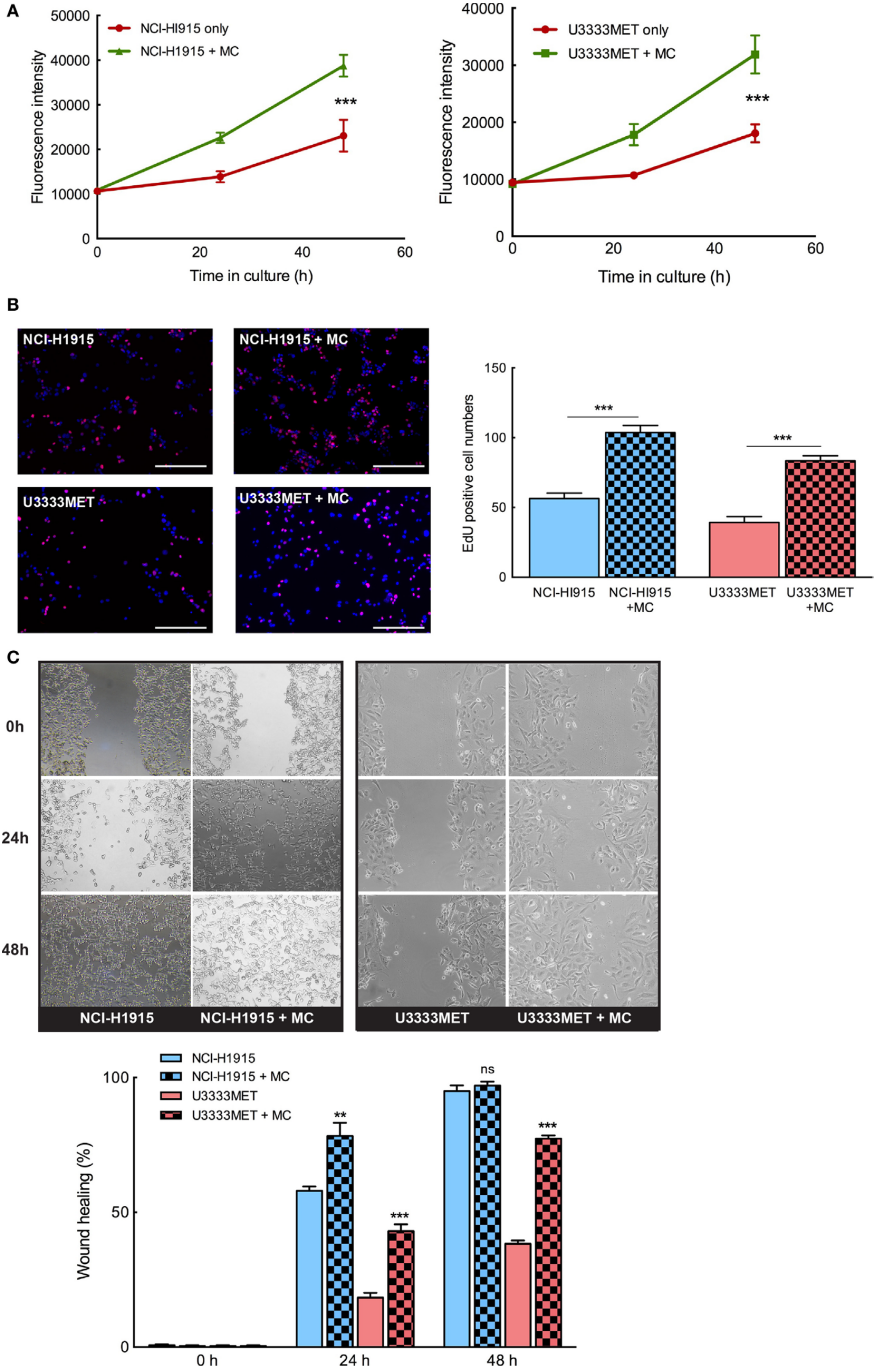
Mast cells (MCs) can significantly induce proliferation and invasion of brain metastasis (BM) cells. The BM cell lines were cocultured with MCs or cultured alone for 24 and 48 h and **(A)** AlamarBlue cell proliferation assay performed with NCI-H1915 (left panel) and U3333MET (right panel) cultured alone (red) or in coculture with LAD2 cells (green). **(B)** EdU was added to the cultures 4 h before the experiment endpoint and the cells were processed for immunofluorescence to analyze incorporated EdU (left panel). The results are expressed as EdU positive cell numbers (right panel). **(C)** Migration capacity of the BM cells was assessed by wound healing assay after 24 and 48 h when grown alone or in coculture with MCs (left panel). The results are shown as percentage of healed wound area (right panel). The experiments were performed three times in triplicates and mean values + SEM was plotted, ns, not significant; **p < 0.01, ***p < 0.001.

In order to evaluate the potential role of MCs in BM, we need to understand the mechanistic interplay between the cells and how this governs the metastases tissue microenvironment. The tumor cells, that cross the BBB hurdle and survive, attempt to lure the brain immune system and proliferate in the new microenvironment (25). We examined the proliferation doubling time (PDT) of the BM cells to evaluate their growth rate, both in the presence and absence of MCs. NCI-H1915 cells (PDT = 48 h) had a relative fast growth rate when compared to the U3333MET cells (PDT = 72 h). But we observed a significant induction in growth rate in both the cell lines when grown in coculture with MCs (Figures 3A,B). We then performed wound healing assay to ascertain the migration efficacy of the BM cell lines. Given the slower growth rate of U3333MET cells, the wound healing time required for them in the absence of MCs was longer than for NCI-H1915 cells that closed the opening almost at 36 h. The migration was increased in both cell lines upon MC coculture (Figure 3C). Although this increase of motility was evident in NCI-H1915 cells, it was statistically significant only for the U3333MET cells at both time points, which we attribute to the faster proliferation rate of NCI-H1915 cells. We hence performed a shorter time point (less than 48 h) migration study for the NCI-H1915 cells to evaluate the migration without taking into consideration the proliferation. Indeed, we could see a significant effect of MC coculture on the migration rate at shorter time points (Figure S1 in Supplementary Material). These observed effects provide evidence that MCs have a positive effect on the growth and propagation of the BM cells.

### Patient-Derived BM Cells and NCI-H1915 Cells Exhibit Enhanced Self-renewal Capacity upon MC Stimulation

**Figure 4:**
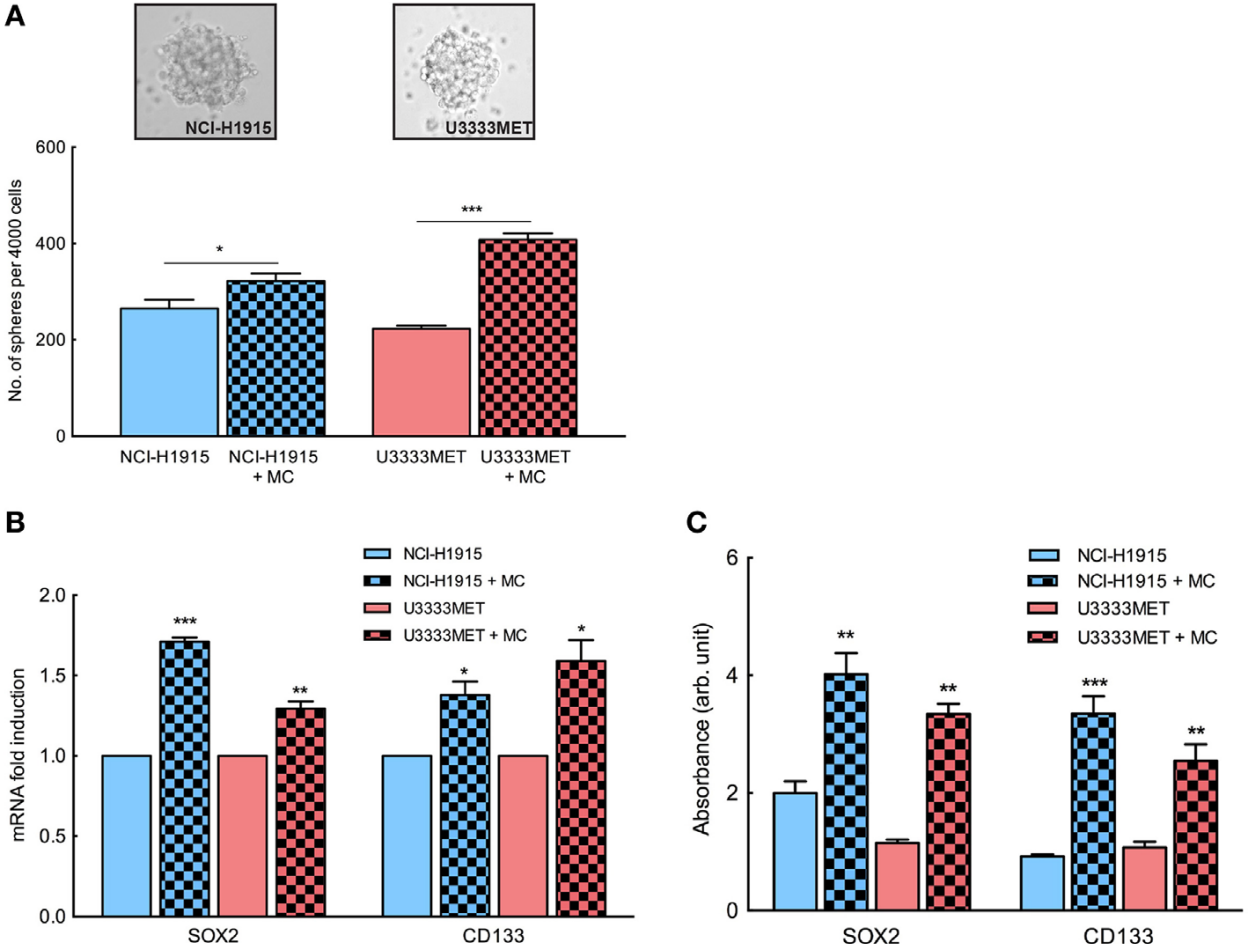
Enhanced self-renewal capacity in brain metastasis (BM) cells upon mast cell (MC) stimulation. BM cells were subjected to tumorsphere formation assay in order to examine the self-renewal stem-like cell population in the BM cells and the influence of MC mediators after coculture. **(A)** Tumorsphere formation assay of patient-derived BM cells U3333MET and the BM cell line NCI-H1915 (upper panel). Sphere forming capacity of the cells was compared before and after coculture with MCs by counting the number of formed spheres. Results are presented as number of spheres formed per 4,000 seeded cells (lower panel). **(B)** Expression analysis of stem cell markers in the BM cells before and after coculture with MCs. β-actin expression level was used for normalization. For each gene, the expression level in the control cells was considered as 1 and the expression levels after coculture was calculated relative to it. **(C)** In-cell ELISA to evaluate SOX2 and CD133 expression in BM cells before and after coculture with MCs. All the experiments were performed three times in triplicates and mean values + SEM was plotted, ns, not significant; **p < 0.01, ***p < 0.001.

Several studies have shown that the presence of tumor-initiating cell populations has a strong correlation with the development of metastases (26, 27). Given the fact that BM is thought to develop from a limited primary tumor cell population, we assessed the self-renewing stem-like population in the BM cell cultures by performing tumorsphere formation assay. NCI-H1915 cells have previously been shown to be capable of forming spheres and retaining self-renewal property. In our study, both patient-derived U3333MET cells and NCI-H1915 cells (Figure 4A) formed spheres. We then assayed the potency of MC mediators by growing both cell lines in coculture with MCs for 48 h and then applying them for tumorsphere assay. There was no significant difference in sphere forming capacity between control NCI-H1915 and U3333MET cells. However, upon MC pretreatment, both cell lines showed a significant increase in sphere formation (Figure 4A). This was also supported by increased expression of cancer stem cell (CSC) markers such as SOX2 and CD133 in U3333MET cells after 48-h coculture with MCs in comparison to control cells (Figure 4B). The increase of CSC markers in the patient-derived U3333MET cells as well as in NCI-H1915 cells was observed both at the gene expression level and at the protein expression level (Figure 4C) making it evident that the presence of MCs in BMs could be beneficial for the tumor-initiating CSCs to establish themselves and propagate. Considering that the two cell lines are from two different patients, we did observe a difference in the effects upon MC coculture between them, indicating cell heterogeneity and individualistic difference in the effect exerted on BM cells by MC mediators.

### Identification of Candidate Genes in BM Cells upon Coculture with MCs

**Figure 5:**
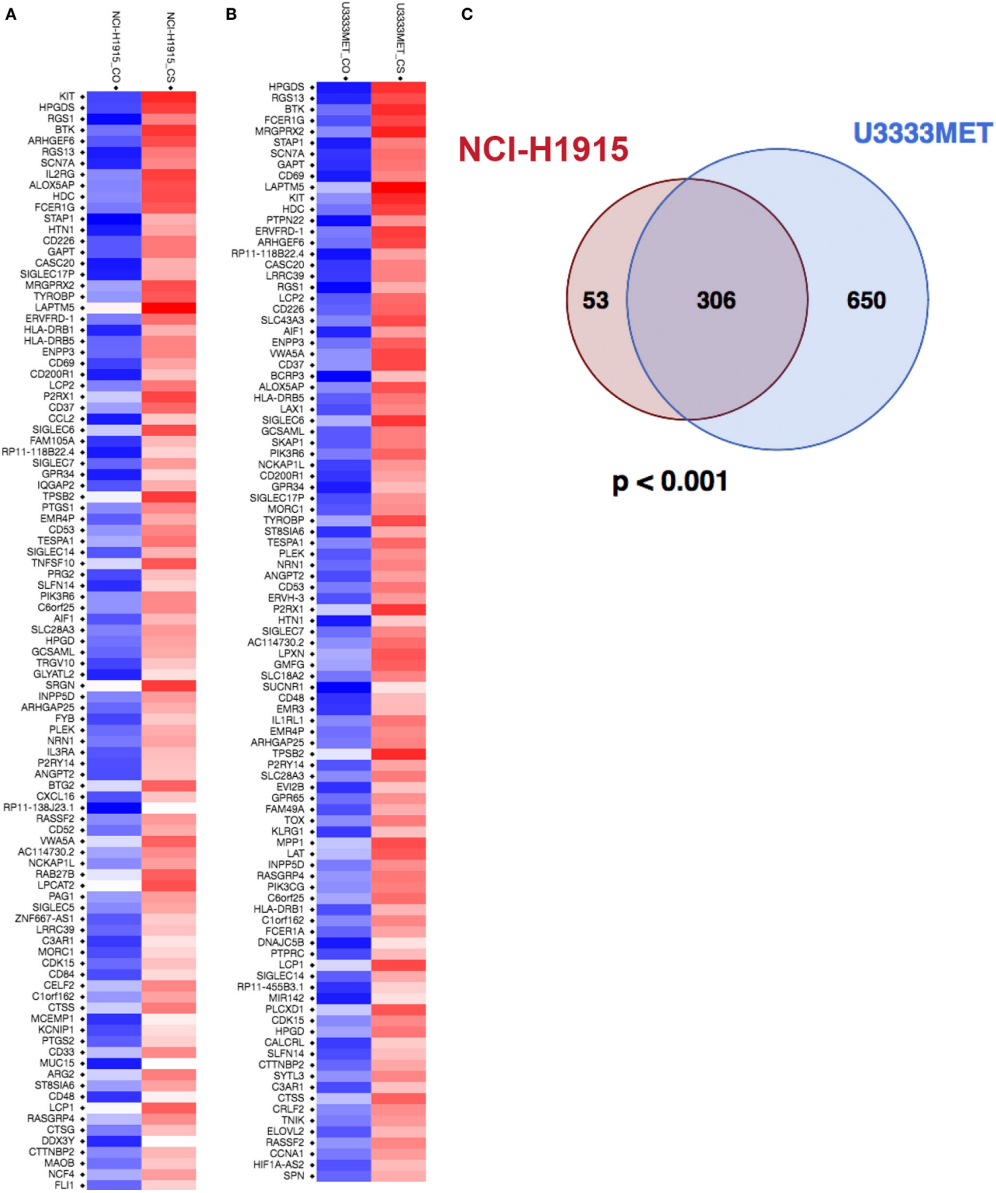
Mast cells (MCs) induce significant changes in expression levels of genes associated with brain metastasis cells. **(A)** Heatmap of the top 100 differentially expressed genes (DEGs) in NCI-H1915 cells after MC coculture. **(B)** Heatmap of the top 100 DEGs in U3333MET cells after MC coculture. **(C)** Venn diagram comparing the DEGs between NCI-H1915 cells and U3333MET cells (***p < 0.001).

To identify potential MC-regulated mediators of tumor growth and metastasis, we performed a global gene expression analysis of the BM cells before and after MC coculture. U3333MET, a patient sample of BM (lung as primary cancer origin) and NCI-H1915 cells, was grown for 48 h with or without indirect MC coculture and then subjected to microarray analysis and subsequent comparative analysis. A principal component analysis of the data showed BM and BM-MC RNA expression to be differentially clustered (Figure S2 in Supplementary Material), indicating that MC mediators have an impact on the BM cells pathophysiology. The entire expression data were subsequently used for GSEA and observed enrichment related to MC activation; IL-8/CXCR1/CXCR2 pathway signaling and immune effector processes were some of the physiological pathways that were of interest (Figure S3 in Supplementary Material). The GSEA also showed that U3333MET cells showed a better enrichment status in comparison to NCI-H1915 cells. Subsequently, the expression level was set at a twofold difference cutoff and the filtered genes were then subjected to further analysis. With the set cutoff, the global gene transcription analysis identified 408 DEGs in MC cocultured NCI-H1915 cells with a significantly altered expression (*p* < 0.05). After eliminating the “LOC” genes, i.e., gene names starting with LOC and with unknown function, 359 DEGs were detected (Figure 5A; Figure S4A in Supplementary Material). While for MC cocultured U3333MET cells about 990 DEGs at a twofold difference including the LOC genes were observed. After discarding the LOC genes and the non-annotated genes, 956 DEGs with more than twofold difference were listed (Figure 5B; Figure S4B in Supplementary Material). All genes in the two lists were subsequently used for the downstream gene ontology and functional annotation clustering on the DAVID platform (Tables S4 and S5 in Supplementary Material). A number of biological modules with a significant enrichment score (ES); e.g., “Transmembrane,” “Glycoprotein,” “Signal” (ES = 7.21); “Inflammation” (ES = 4.38), “Cell Adhesion” (ES = 4.36), and “Integrin Mediated Signaling” (ES = 3.2) are among the top identified clusters and involved in tumorigenesis. Considering that the two BM cell lines were from different patients but had the same primary cancer origin and demonstrated distinct differential gene set expression upon MC coculture, we examined whether a similarity pattern between them existed. Indeed, a significant overlap of the DEGs (Figure 5C) was observed. To ascertain the biology behind these 306 common genes, we subjected them to pathway analysis to compute overlaps with publicly available datasets and significant overlap was observed with gene sets related to oncogenic signature, cancer module, molecular function, biological process, and transcription factor targets (Figures S5 and S6 in Supplementary Material).

The ESs and identification of biological modules involved in regulation of signaling pathways, inflammatory response, and cellular functionality suggest an efficient MC–BM cell cross talk.

### MCs Support BM Cells Propagation *via* Secretion of IL-8, IL-10, VEGF, and MMP2

**Figure 6:**
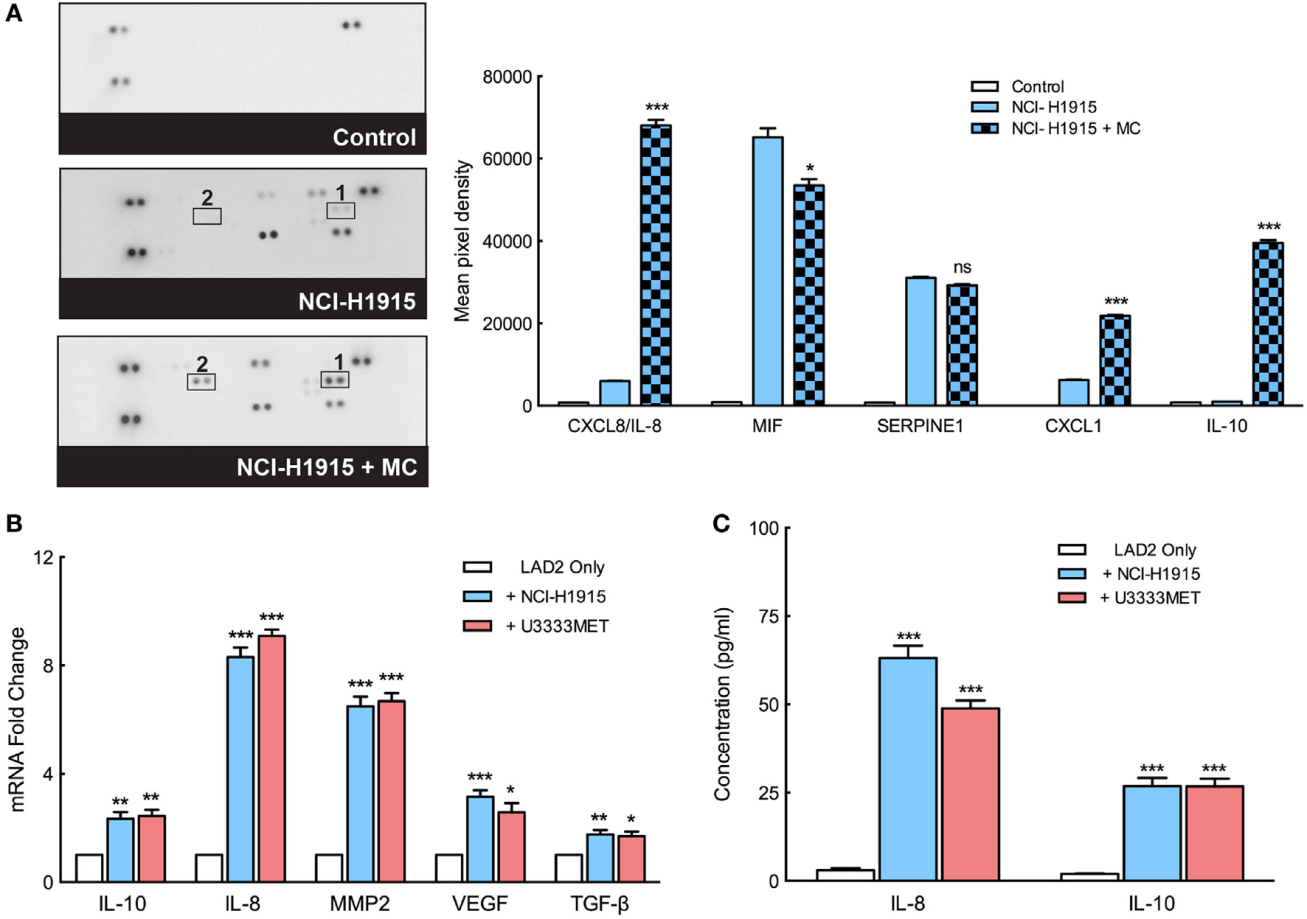
Mast cells (MCs) secrete angiogenic and tumorigenic mediators to support immune escape and progression of metastases. **(A)** Left panel: the secreted cytokine profiles of supernatant from MCs cultured in control medium, in coculture with NCI-H1915 or NCI-H1915 cultured in control medium. Right panel: quantification of the dots of interest is plotted as integrated pixel intensities. **(B)** qPCR analysis for the expression of IL-10, IL-8, matrix metalloprotease 2 (MMP2), vascular endothelial growth factor (VEGF), and TGF-β in LAD2 cells before and after coculture with brain metastasis (BM) cells. β-actin expression level was used for normalization. For each gene, the expression level in the control cells was considered as 1 and the expression levels after coculture was calculated relative to it. **(C)** Levels of IL-8 and IL-10 secreted by LAD2 cells (before and after coculture with the BM cells) were measured by ELISA. All the experiments were performed three times in triplicates and mean values + SEM was plotted, ns, not significant; **p < 0.01, ***p < 0.001.

We found that BM cells thrive in the presence of MCs and even show a better self-renewal capacity, characteristics that indicate their success in immune escape, and progression of metastasis. MCs, therefore, seem to play a role in setting up the platform for BM cell intravasation. Indeed, a cytokine array analysis of mediators secreted by MCs before and after MC–BM cell coculture shows an induction of a number of cytokines (Figure 6A). A significant increase in gene expression of IL-8, IL-10, VEGF, and MMP2 in the MCs was observed after coculture with BM cells (Figure 6B). Elevated levels of secreted IL-8 and IL-10, as observed in the cytokine array, were even confirmed by ELISA that demonstrated significant increase of them after coculture with BM cells (Figure 6C). These factors predict poor outcome in cancer by supporting angiogenesis, cell growth, infiltration of M2 macrophages, and recruitment of immunosuppressive T cell population (28–30). Secreted CXCL1 can bind to ligands expressed on BM cells and mediate a signaling network to support the BM cell growth, promote their self-renewal capacity, and eventually confer resistance to chemotherapy that often coincides with cancer metastasis.

### MC Infiltration in BM Relates to MC Presence in Matched Primary tumors

**Figure 7:**
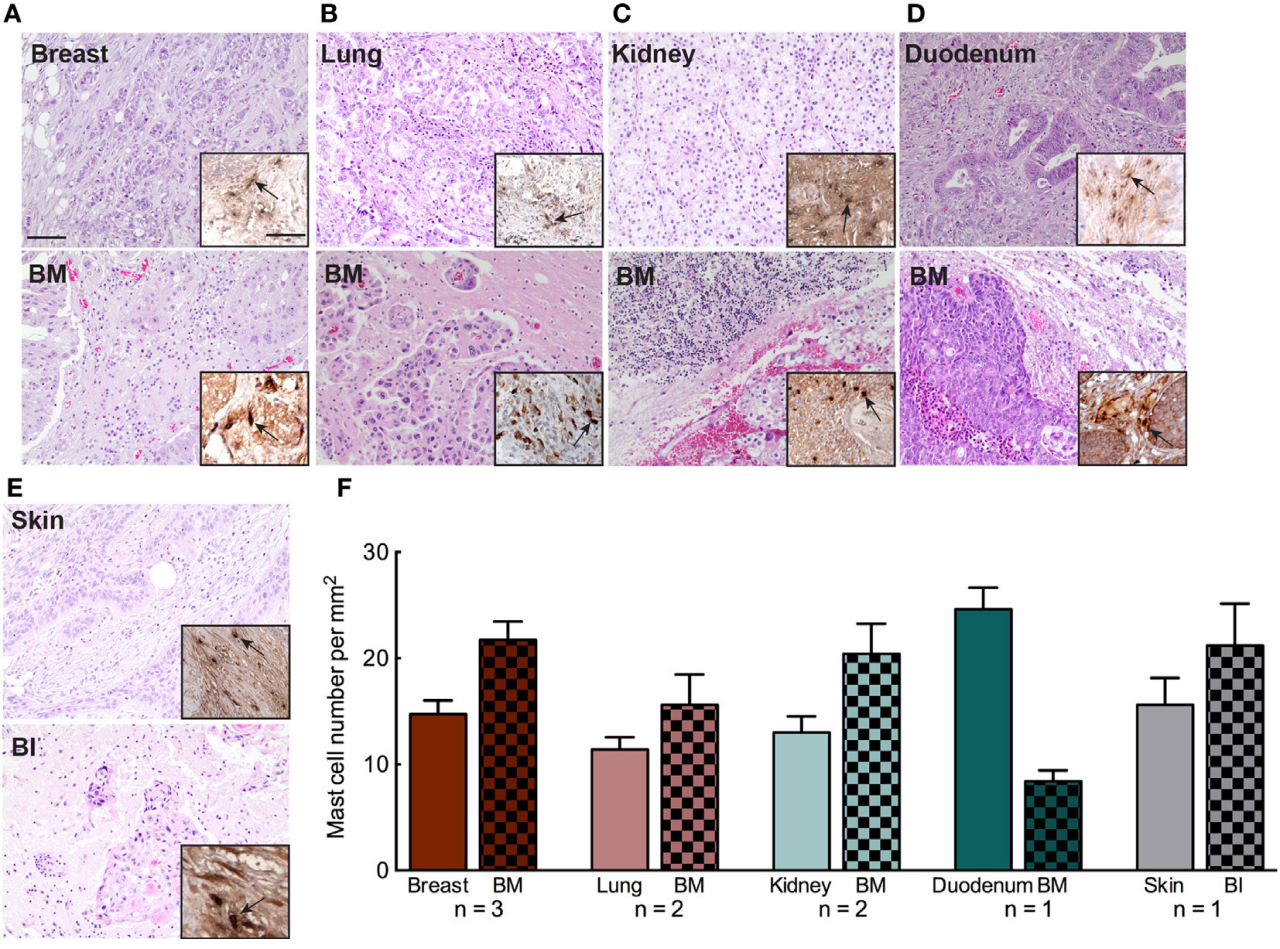
Mast cell (MC) infiltration in primary tumor microenvironment of patients with brain metastasis (BM). **(A–E)** Representative pictures of H&E staining of primary cancer tissue and patient matched BM tissue. Upper panel: breast, lung, kidney, duodenum, and skin. Lower panel: patient matched BM tissue. The patient with skin cancer **(E)** was evaluated to have brain invasion (BI). Insets to the right in each picture: IHC staining for MC tryptase showing MC infiltration in BM patient’s primary cancer (upper panel) and BM tissue (lower panel). Pictures are taken at 20× magnification. **(F)** Quantification of MC infiltration in primary cancer tissue and the corresponding BM tissue.

Mast cell infiltration in primary tumor has been correlated to enhance dissemination, extravasation, and metastasizing capacity of the cancer cells. Hence, the presence of MCs in primary tumors can be indicative of future metastasis. We also screened for the level of MC infiltration in the primary tumor tissue of nine patients diagnosed with BM (Table S3 in Supplementary Material). Primary tumor tissues and their matched BM tissues of the patients were evaluated by H&E staining followed by MC tryptase staining. We observed that patients with MC infiltrated BM had abundant number of MCs in their primary tumors as well (Figures 7A–F). However, we were not able to perform any statistical analysis due to the lack of adequate number of samples that were available. Hence, larger cohorts of primary tumors and matched BM are required to ascertain whether this observation is conclusive. Furthermore, the results should be compared with primary tumors that have not metastasized to the brain. Our data support the notion that tumor cells from a MC-rich primary cancer microenvironment are probably potent and better equipped to survive immune surveillance and establish in another location to form metastases.

## Discussion

Metastatic cancer to the brain shows a poor prognosis and poses a severe clinical problem due to the lack of effective therapies and knowledge of mechanisms that control metastatic growth in the brain. BM is a major cancer in the CNS with lung cancer, breast cancer, and melanoma accounting for most clinical cases of BM from non-CNS primary tumors (31, 32). BM usually manifest at late stages of metastatic disease progression, although this latency varies among different primary tumor types and some may already exhibit metastatic lesions in the CNS at the time of primary tumor diagnosis (33), whereas others might take years after regression of primary tumor (34, 35). Distinct primary tumor cell properties are critical factors responsible for the discrepancy in BM latency, although the exact molecular mechanism remains unclear.

Immune escape being acknowledged as a hallmark of cancer and with the CNS considered as immune privileged, the capacity of inflammatory response in the brain seem to be limited. Indeed, the knowledge about the inflammatory microenvironment of BM is still elusive. However, in spite of the BBB, immune cell infiltrations have been observed in BM with a tendency to enable the cancer cell to colonize. Infiltrating tumor-associated macrophages (TAMs) have been shown to have a metastasis promoting function by enhancing cancer cell intravasation, whereas TILs seem to have opposing effects (5, 8, 36). In this study, we screen for MCs in a large cohort of BM samples and provide evidence for MC as a participant in BM inflammation. The current study is the first to demonstrate MC infiltration in BM in a large patient cohort. In our *in vitro* coculture experiments, BM cells significantly induced MCs to secrete IL-8 and IL-10, two cytokines that have been repeatedly reported to be involved in shaping and supporting the metastatic cascade. The role of IL-8 in the tumor microenvironment is profound, it promotes tumor cell invasion and migration, induces TAMs to secrete factors that further increase the cell proliferation and invasion at the tumor site (37). IL-8 posses a unique autoregulatory property, e.g., secretion of IL-8 can activate an autocrine feedback loop to activate neighboring endothelial cells for further secretion. This eventually serves to maintain the BM cells in a mesenchymal state, and also induce neighboring cells to proliferate and invade. IL-10 in cancer has been reported to exhibit differential effects that seem to be contradictory in some cases. Some of its well-studied role in pathogenesis and tumorigenesis includes stimulation of regulatory T cells, attenuation of cytokine production, proliferation, and migratory capacities of effector T cells, eventually leading to an immunosuppressive tumor microenvironment (29, 38). BM cell-induced secretion of IL-10 from MCs can attribute tumor-specific immune surveillance in the tumor microenvironment and modulate cell growth and extracellular matrix (ECM) remodeling.

Further, we observe an increased expression of VEGF by MCs in response to BM cell stimulation. VEGF can promote neoangiogenesis in the tumor tissue thereby supporting BM growth from micro- to macrometastases, which had been clinically shown to be counteracted by the use of VEGF antibody (39, 40). In addition to supporting angiogenesis (28), VEGF from MCs can increase vascular permeability in the BM tissue and help in better mobilization of cancer cells to the tissue. BM cells can also promote the expansion of myeloid-derived suppressor cells (MDSCs) within the tumor microenvironment by inducing secretion of VEGF from MCs that in turn can activate JAK2/STAT3 signaling in myeloid progenitor cells (41). Subsequently, these MDSCs can then become activated by other factors such as IFN-γ and TGF-β.

Mechanisms whereby MMPs influence tumor behavior have been mainly attributed to the proteolytic ability of MMPs to degrade ECM proteins. It thereby modulates the relationship between tumor cells and host tissue stroma. In our functional study, we observe an increase in proliferation and migration capacity of the BM cells, which is associated with simultaneous induction of MMP2 secretion from MCs. An enrichment of genes in BM cells involved in transmembrane protein functionality was observed upon MC coculture, along with an induction of EGFR (microarray data), involved in activation of the MAPK pathway and upregulation of CDK6, a major regulator of cell cycle. Our results suggest that MC contribution in BM development is partly mediated *via* IL-8 and MMP2 by activation of the MAPK pathway (Figure S7 in Supplementary Material). Inter- and intratumor heterogeneity has been documented in metastatic brain tumors (42, 43). However, the genomic basis for the development of metastases to the brain from the primary tumor and the extent to which BM shares the genetic profile of the primary tumor still remains unclear (44). Global gene expression profiling has been extensively used to identify potential genes regulating tumor growth establishment. The global transcription analysis of the patient-derived U3333MET cells and the NCI-H1915 cells grown alone or in coculture with MCs, allowed us to evaluate how the vicinity of MCs affects BM cell functionality. A significant overlap of the 306 DEGs from the two cancer cell lines clearly demonstrates an effective MC-BM cells cross talk. However, an intertumoral heterogeneity between the two cell lines is also observed with over 650 totally different DEGs in U3333MET cells and 53 DEGs in NCI-H1915 cells after MC coculture (Figure 5C). The efficiency of MC-BM cell cross talk seems to be more potent for the relatively slow growing U3333MET cells compared to the more aggressive NCI-H1915 cell line. The enrichment analysis also demonstrates activation of the IL-8/CXCR1/CXCR2 pathway in the BM cells that are crucial for the activation of multiple intracellular signaling pathways to regulate proliferation, differentiation, and migration of tumor cells. It can also initiate a tumor immunosuppression cascade in the BM microenvironment, thereby making the tumor resistant to treatment.

The inflammatory tumor microenvironment has been under focus in the recent years and has been target for prognostic or therapeutic significance in various malignancies. Immunomodulatory drugs have shown remarkable and lasting responses in several tumor types, but their feasibility, as treatment target for BM needs to be ascertained. Brain metastatic tumors have so far hardly succumbed to conventional chemotherapy and targeted therapeutic treatments, partly due to the inability of the drugs to penetrate the BBB. Therefore, current standard treatments still include surgery and radiosurgical procedures. MCs represent an ideal candidate for targeted therapy. They can cross the BBB to infiltrate the BM environment and deliver specific mediators. This makes them perfect carriers for engineered site-specific delivery of immunostimulatory and/or tumor suppressing mediators in BM.

On the other hand, in order to suppress the negative effect of MCs in BM, MC stabilizers might be used as potential therapeutic agents to prevent MC activation. One such candidate could be the well-studied MC stabilizer, disodium cromoglycate (cromolyn), which has been previously shown to be capable of increasing BBB stability (45, 46) and to have antitumor effects as well (47, 48).

In summary, this is the first report demonstrating MC infiltration and their role in BM. Although single-handedly MCs are unlikely to impact durable responses in expansive refractory BMs, our results strongly support the MC mediator’s effect on sustenance and induction of the metastatic capacity of the BM cells. Finally, the present findings warrant further investigations on the role of MCs in metastatic cancer growth in the brain, with the aim to characterize the MC-dependent metastatic pathways and to identify novel drug targets.

## Ethics Statement

This study involving human tissue samples was approved by the Ethics Committee of Uppsala University (Dnr 2014/535) and written informed consent was solicited prior to collection of the samples. Informed consent for the use of human brain tissue and for access to medical records for research purposes was followed as per ethical permission, and all material obtained in compliance with the Declaration of Helsinki. All human tissue samples (Table S1 in Supplementary Material) were obtained from Uppsala Biobank material. All human tumor samples have been evaluated based on the WHO classification by experienced neuropathologist.

## Author Contributions

AR and ET contributed to the conception and design of research; ET, LU, GH, FP, and IA contributed in the conceptual and practical establishment of the research; AR, SL, and IG performed experiments and analyzed data; AR, SL, HW, ET, and IA analyzed and interpreted the data; AR, ET, HW, FS, LU, and IA participated in the writing and revision of the manuscript. All authors read and approved the final version of manuscript.

## Conflict of Interest Statement

The authors declare that the research was conducted in the absence of any commercial or financial relationships that could be construed as a potential conflict of interest. The reviewer, EC, and the handling editor declared their shared affiliation, and the handling editor states that the process nevertheless met the standards of a fair and objective review.
