# Supplementary material for: Mast Cell Infiltration in Human Brain Metastases Modulates the Microenvironment and Contributes to the Metastatic Potential
Source: Front Oncol. 2017 Jun 2;7:115. doi: 10.3389/fonc.2017.00115 (PMC5454042; doi:10.3389/fonc.2017.00115)
Supplement: Supplementary file 1 [file Table_1.DOCX]

| **Patient no.** | **Gender** | **Metastasis tissue diagnosis** | **Metastasis tissue** | **Primary tumor** | **Metastases operation** | **Age (years)** | **Operation type** |
| --- | --- | --- | --- | --- | --- | --- | --- |
| 1 | F | Metastasis adenocarcinoma | Brain | Breast | 2010 | 68 | Surgery |
| 2 | F | Metastasis adenocarcinoma | Brain | Breast | 2010 | 77 | Surgery |
| 3 | F | Metastasis adenocarcinoma | Brain | Breast | 2010 | 57 | Surgery |
| 4 | F | Metastasis adenocarcinoma | Brain | Breast | 2010 | 76 | Surgery |
| 5 | F | Metastasis adenocarcinoma | Brain | Breast | 2011 | 65 | Biopsy |
| 6 | F | Metastasis adenocarcinoma | Brain | Breast | 2012 | 60 | Surgery |
| 7 | F | Metastasis adenocarcinoma | Brain | Breast | 2012 | 63 | Surgery |
| 8 | F | Metastasis adenocarcinoma | Brain | Breast | 2013 | 51 | Biopsy |
| 9 | M | Metastasis renal cell carcinoma | Brain | Kidney | 2013 | 75 | Biopsy |
| 10 | M | Metastasis renal cell carcinoma | Brain | Kidney | 2013 | 56 | Surgery |
| 11 | F | Metastasis adenocarcinoma | Brain | Kidney | 2012 | 64 | Surgery |
| 12 | M | Metastasis adenocarcinoma | Brain | Kidney | 2012 | 65 | Surgery |
| 13 | M | Metastasis renal cell carcinoma | Brain | Kidney | 2011 | 78 | Surgery |
| 14 | M | Metastasis adenocarcinoma | Brain | Lung | 2013 | 70 | Surgery |
| 15 | M | Metastasis adenocarcinoma | Brain | Lung | 2012 | 63 | Post mortem |
| 16 | M | Metastasis adenocarcinoma | Brain | Lung | 2012 | 61 | Surgery |
| 17 | F | Metastasis adenocarcinoma | Brain | Lung | 2012 | 59 | Surgery |
| 18 | F | Metastasis adenocarcinoma | Brain | Lung | 2011 | 68 | Surgery |
| 19 | M | Metastasis adenocarcinoma | Brain | Lung | 2011 | 67 | Surgery |
| 20 | M | Metastasis adenocarcinoma | Brain | Lung | 2011 | 67 | Surgery |
| 21 | M | Metastasis adenocarcinoma | Brain | Lung | 2010 | 66 | Surgery |
| 22 | F | Metastasis adenocarcinoma | Brain | Lung | 2010 | 60 | Post mortem |
| 23 | M | Metastasis adenocarcinoma | Brain | Lung | 2011 | 50 | Surgery |
| 24 | F | Metastasis adenocarcinoma | Brain | Colon | 2012 | 45 | Surgery |
| 25 | M | Metastasis adenocarcinoma | Brain | Colon | 2012 | 69 | Surgery |
| 26 | M | Metastasis adenocarcinoma | Brain | Colon | 2012 | 61 | Surgery |
| 27 | M | Metastasis adenocarcinoma | Brain | Colon | 2011 | 56 | Surgery |
| 28 | F | Metastasis adenocarcinoma | Brain | Colon | 2011 | 75 | Surgery |
| 29 | F | Metastasis adenocarcinoma | Brain | Colon | 2011 | 55 | Surgery |
| 30 | M | Metastasis adenocarcinoma | Brain | Colon | 2010 | 82 | Surgery |
| 31 | M | Metastasis adenocarcinoma | Brain | Colon | 2010 | 49 | Surgery |
| 32 | M | Metastasis adenocarcinoma | Brain | Rectum | 2010 | 52 | Surgery |
| 33 | F | Metastasis adenocarcinoma | Brain | Rectum | 2010 | 59 | Surgery |
| 34 | F | Metastasis adenocarcinoma | Brain | Ovary | 2010 | 58 | Surgery |
| 35 | F | Metastasis adenocarcinoma | Brain | Ovary | 2012 | 68 | Surgery |
| 36 | M | Metastasis adenocarcinoma | Brain | Duodenum | 2011 | 74 | Surgery |
| 37 | F | Metastasis adenocarcinoma | Brain | Fallopian tube | 2010 | 62 | Surgery |
| 38 | F | Metastasis adenocarcinoma | Brain | Peritoneum | 2010 | 64 | Post mortem |
| 39 | F | Metastasis adenocarcinoma | Brain | Uterus | 2012 | 66 | Surgery |
| 40 | M | Squamous carcinoma (invasion) | Brain | Skin | 2010 | 95 | Post mortem |

**Table S1. Patient characteristics and clinical details**
