# Supplementary material for: Mast Cell Infiltration in Human Brain Metastases Modulates the Microenvironment and Contributes to the Metastatic Potential
Source: Front Oncol. 2017 Jun 2;7:115. doi: 10.3389/fonc.2017.00115 (PMC5454042; doi:10.3389/fonc.2017.00115)
Supplement: Supplementary file 2 [file Table_2.DOCX]

| \| **Target gene** \|  \|  \| \| --- \| --- \| --- \| | **Forward sequence** | **Reverse sequence** |
| --- | --- | --- | --- | --- | --- |
| ß ACTIN | GGACTTCGAGCAAGAGATGG | AGCACTGTGTTGGCGTACAG |
| SOX2 | GCGAACCATCTCTGTGGTCT | AAAATGGAAAGTTGGGATCG |
| CD133 | CACTACCAAGGACAAGGCGTTC | CAACGCCTCTTTGGTCTCCTTG |
| VEGF | CTACCTCCACCATGCCAAGT | GCAGTAGCTGCGCTGATAGA |
| IL-8 | ACAGCAGAGCACACAAGCTT | CTGGCAACCCTACAACAGAC |
| TGF-ß | TACCTGAACCCGTGTTGCTCTC | GTTGCTGAGGTATCGCCAGGAA |
| MMP2 | AGCGAGTGGATGCCGCCTTTAA | CATTCCAGGCATCTGCGATGAG |
| TPSAB1 | GTGACGCAAAATACCACCTTGGC | CCATTCACCTTGCACACCAGGG |
| CMA1 | TGTGGGCAATCCCAGGAAGACA | GACCGTCCATAGGATACGATGC |
| CPA3 | ATCGGCACTGACCTCAACAGGA | CTCTTTCTCGGACTCTGGTGCA |
| IL-10 | TCTCCGAGATGCCTTCAGCAGA | TCAGACAAGGCTTGGCAACCCA |

**Table S2: Human primers used for qPCR**
