# Supplementary material for: Mast Cell Infiltration in Human Brain Metastases Modulates the Microenvironment and Contributes to the Metastatic Potential
Source: Front Oncol. 2017 Jun 2;7:115. doi: 10.3389/fonc.2017.00115 (PMC5454042; doi:10.3389/fonc.2017.00115)
Supplement: Supplementary file 3 [file Table_3.DOCX]

| **Patient no.** | **Gender** | **Age** | **Primary tumor operation type** | **Primary tumor** | **Time period to BM operation**  **(year)** | **BM diagnosis** |
| --- | --- | --- | --- | --- | --- | --- |
| 1 | F | 68 | Surgery | Breast | 14 | Metastasis adenocarcinoma |
| 2 | F | 76 | Surgery | Breast | 2 | Metastasis adenocarcinoma |
| 3 | F | 65 | Surgery | Breast | 2 | Metastasis adenocarcinoma |
| 4 | M | 64 | Surgery | Kidney | 7 | Metastasis adenocarcinoma |
| 5 | F | 65 | Surgery | Kidney | 4 | Metastasis adenocarcinoma |
| 6 | M | 67 | Surgery | Lung | 3 | Metastasis adenocarcinoma |
| 7 | F | 60 | Post mortem | Lung | > 1 | Metastasis adenocarcinoma |
| 8 | M | 95 | Surgery | Skin | 2 | Squamous carcinoma (invasion) |
| 9 | M | 74 | Surgery | Duodenum | 5 | Metastasis adenocarcinoma |

**Table S3. Patient characteristics and clinical details**
