# Supplementary material for: Mast Cell Infiltration in Human Brain Metastases Modulates the Microenvironment and Contributes to the Metastatic Potential
Source: Front Oncol. 2017 Jun 2;7:115. doi: 10.3389/fonc.2017.00115 (PMC5454042; doi:10.3389/fonc.2017.00115)
Supplement: Supplementary file 4 [file Table_4.DOCX]

| **Term** | **Benjamini** | **Enrichment Score** | **Genes** |
| --- | --- | --- | --- |
| Transmembrane | 3.9E-7 | 7.21 | ATP1A4,ATP8A2,ATP8B4,ATP2A3,BTK,CCR2, CXCL16,CD200R1,CD226,CD300LF,CD300A,CD33, CD37,CD4,CD48,CD52,CD53,CD69,CD84,CMTM5, DNAJC15,DNAJC5B,ELOVL2,ELOVL7,FCER1A,FCER1G,  GPR137B GPR141, GPR34, GAB3 ,GAPT , JAK3 , KIT  KEL, L1CAM, LMBRD2, MRGPRX2, MAS1L , MFNG MYB,GNPTAB,NCKAP1L,RAB27B,RAB39A,RAB44, RASGRP4,ARHGAP15,ST8SIA6,TIAM1,TYROBP,ADORA3, AIF1,AQP10,ALOX5AP,ALOX5,CALCRL,CASQ1,C1orf162, C6orf25 ,CSF2RA,CSF2RB, C3AR1 , CYSLTR1 ,CRLF2  DOCK2 , EVI2A , EVI2B , ENPP3, ERVFRD-1 , ELMO1 , EPB41, FAM171A1 , FAR2 , GBP2 , HCST , HCLS1 , HAVCR2 , HK2 , IGSF3 , INPP5D , ITGA4 , ITGA9 , ITGAM , IPCEF1, ICAM1 , IFI6 , IL1RAPL1, IL12RB2 , IL2RG , IL3RA, IL9R , KLRG1 ,KSR1, LPXN , LCP1 , LAX1, LPAR5 , LPCAT2, LAPTM5 , MR1, HLA-DPA1 , HLA-DRB1, HLA-DRB3 , HLA-DRB5, MCEMP1 , MRAP2, MAOB , MUC15 NRN1 , NCF4 , NUP210 , OR10H5 , OR13A1 , PREX1, PIK3CG ,PIK3C2B, PIK3R6 , PLA2G2A , PAG1, KCNN1 , KCNIP1 , KCNQ1 , PTGER3, PTGER4 , PTGS1 , PTGS2, PRKCQ , PTPRC , PCDHB5 , PCDHB6 , P2RX1 , P2RY14 , RIPK3 , RGS1 , RGS16, RNF122 , RNF125 , RNF130, SELL, SELPLG, SEMA4D, SGK1, SIGLEC14 , SIGLEC5 , SIGLEC6, SIGLEC7, SIGLEC8, SIGLEC9, SPN, SLAMF1, SMIM3, SCN7A, SLC17A7, SLC18A2 SLC24A3, SLC28A3, SLC43A3, SLC45A3, SLC46A3, SLCO2B1, SYK, SKAP1  SUCNR1, STX3, TESPA1, TMEM150C, TNFSF10, UNC13D |
| Signal | 2.6E-3 | 7.21 | ATP8B4, CCL1, CCL2, CCL4L2, CCL5, CXCL16, CD200R1, CD226, CD300LF, CD300A, CD33,CD4,CD48,CD52,CD84  FCER1A, FCER1G, KIT, L1CAM, MAS1L TRAV8-3, TRGV5, TYROBP, ANGPT2, CALCRL, CASQ1, CTSG, CTSS, CTSW, C6orf25, CSF2RA, CSF2RB, CRLF2, EVI2A, EVI2B, ERVFRD-1, FAM171A1, GDF2, HCST, HAVCR2, HTN1, IGSF3, ITGA4, ITGA9, ITGAM, ICAM1, IFI6, IL1RAPL1, IL12RB2, IL2RG, IL3RA, IL9R, LTBP1, MR1, HLA-DPA1,  HLA-DRB1, HLA-DRB3, HLA-DRB5, MANBA, MUC15, NRN1, NUP210, PLA2G2A, PTGS1, PTGS2, PTPRC, PRG2  PCDHB5, PCDHB6, RNASE6, RNF130, SFRP1, SELL, SELPLG, SEMA4D, SRGN, SERPINI1, SIGLEC14  SIGLEC5, SIGLEC6, SIGLEC7, SIGLEC8, SIGLEC9, SPN, SLAMF1, SLC24A3, SLC46A3, SLCO2B1, TRANK1  TCN1, TPSB2, UTS2 |
| Glycoprotein | 2.7E-7 | 7.21 | CCL2, CD226, CD300A, CD33, CD4, L1CAM, MTSS1, ITGA4, ITGA9, ITGAM, ICAM1, LPXN, PRKX, PCDHB6, SELL, SELPLG, SEMA4D, SIGLEC14, SIGLEC5, SIGLEC6, SIGLEC7, SIGLEC8, SIGLEC9, SLAMF1 |
| Inflammation | 1.4E-5 | 4.38 | BTK, CD84, FCER1G, ITK, JAK3, PYCARD, TYROBP, HAVCR2, IFIH1, KLRG1, MR1, PIK3CG, SIGLEC14, SLAMF1, SYK, FCER1A, FCER1G, MAP3K1, NFATC2, VAV1, INPP5D, PLCG2, PTPN6, VAV3 |
| Cell Adhesion | 6.3E-4 | 4.36 | CCL2, CD226, CD300A, CD33, CD4, L1CAM, MTSS1, ITGA4, ITGA9, ITGAM, ICAM1, LPXN, PRKX, PCDHB6, SELL, SELPLG, SEMA4D, SIGLEC14, SIGLEC5, SIGLEC6, SIGLEC7, SIGLEC8, SIGLEC9, SLAMF1 |

**Supplementary Table S4.** Annotation clustering on DAVID platform of enriched gene sets in the NCI-H1915 cells after MC co-culture.
