# Supplementary material for: Mast Cell Infiltration in Human Brain Metastases Modulates the Microenvironment and Contributes to the Metastatic Potential
Source: Front Oncol. 2017 Jun 2;7:115. doi: 10.3389/fonc.2017.00115 (PMC5454042; doi:10.3389/fonc.2017.00115)
Supplement: Supplementary file 5 [file Table_5.DOCX]

| **Term**  **(Annotation cluster)** | **Benjamini** | **Enrichment Score** | **Genes** |
| --- | --- | --- | --- |
| Membrane, Transmembrane | 3.0E-10 | 7.84 | BCA12, ABCC2, ABCC3, ATP6V0A2, ATP1A4, ATP8A1  ATP8A2, ATP8B4, ATP9A, BNIP3L, CCR2, CD2, CD200R1, CD22, CD226, CD300LF, CD300A, CD33, CD37, CD48, CD52  CD53, CD69, CD82, CD84, CHML, CMTM5, DPY19L2P1  DNAJC15, ELOVL2, F2RL3, FASLG, FCER1A, FCER1G  FCGR2A, FCGR2B, GPR135, GPR141, GPR162, GPR34  GPR65, GPR85, GAB3, GAPT, KIAA0319, KIT, KEL, L1CAM  MRGPRX2, MAS1L, MFNG, MYB, GNPTAB, ASAH1, NDST2  NCKAP1L, ORAI3, ST3GAL5, ST8SIA4, ST8SIA6, STEAP1  STEAP2, TNFRSF14, TYROBP, XKR6, YME1L1, ZFP14, ABHD6, ACSL4, ADORA3, AMIGO2, ADRA1D, ADRB2  ACER3, AMHR2, AQP10, ALOX5AP, B4GALNT3, BMPR1A  CDH6, CDH7, CALCRL, CAMLG, CACNA2D1, CHST12  CPM, CEACAM1, CLIC6, CLCN6, C1orf162, C16orf54, C6orf25, F8, CSF2RA, CSF2RB, C3AR1,CR1, CNST, CPOX  CYSLTR1, CTNS, CYB561D2, CRLF2, DGAT1, DRD2, EVI2A  EVI2B, ENPP3, ERVFRD-1, EGFR, ERMAP, EPB41, EPOR  EXTL3, FMO4, FLT3LG, FLT3, GABRB2, GABRE, GDPD1  HCST, HAVCR2, HRH4, HILPDA, ITPR1, IGF1R, ITGA2, ITGA4, ITGA6, ITGA9, ITGAL, ITGAM, ITGAV, ITGB4  ITGB6, ICAM3, IFITM2, IL1RAPL1, IL1RL1, IL12RB1, IL12RB2, IL18R1, IL18RAP, IL3RA, IL5RA, IL9R, KLRG1  LILRA2, LAT, LINC00493, LAX1, LPAR5, LPCAT2, LAPTM5  MFSD6L, MR1, HLA-DPA1, HLA-DRB1, HLA-DRB3  HLA-DRB5, MAL2, MAN1A1, MCEMP1, MS4A2, MS4A4E  METTL7A, MAOB, MUC15, MUC16, MCTP1, MCTP2  MYOF, NRROS, NBAS, NETO2, OCLN, OR1L3, OR1L8  OR10H5, OR13A1, OR14K1, OR2AK2, OR2L13, OR2L2  OR2M3, OR2M7, OR5B21, PMP22, PLN, PAG1, PLXNA2  KCNMA1, KCNN1, KCNQ1, PTGER3, PMEPA1, PROS1  PTPRC, PTPRK, PCDHB5, PCDHB6, P2RX1, P2RX4, P2RY1  P2RY14, RNF122, RNF130, RNF144A, RYR2, SELL, SELPLG  SIGLEC14, SIGLEC5, SIGLEC6, SIGLEC7, SIGLEC8, SIGLEC9, SPN, SFXN3, SLAMF1, SMIM3, SCN3A, SCN7A  SCN9A, SLC10A5, SLC17A7, SLC18A2, SLC2A3, SLC22A3, SLC24A3, SLC25A30, SLC27A2, SLC28A3, SLC35F1, SLC39A11, SLC4A4, SLC43A3, SLC45A3, SLC46A3, SLC5A12, SLC6A9, SLC7A11, SLC8A3, SLC9A3, SLCO2B1  SGPP1, STRA6, SUCNR1, SYT11, STX3, STXBP2, TBXAS1  TRPV2, TMC8, TMEM135, TMEM140, TMEM145, TMEM150C, TMEM154, TMEM173, TMEM55A  TPSG1, TNFSF10 |

**Supplementary Table S5.** Annotation clustering on DAVID platform of enriched gene sets in the U3333MET cells after MC co-culture.

| Glycoprotein | 3.5E-7 | 7.84 | CD22, CD226, CD300LF, CD300A, CD33, CD37, CD48, CD52  CD53, CD69, CD82, CD84, CHML, CMTM5, DPY19L2P1  DNAJC15, ELOVL2, F2RL3, FASLG, FCER1A, FCER1G  FCGR2A, FCGR2B, GPR135, GPR141, GPR162, GPR34  GPR65, GPR85, GAB3, GAPT, KIAA0319, KIT, KEL, L1CAM  MRGPRX2, MAS1L, MFNG, MYB, GNPTAB, ASAH1, NDST2  NCKAP1L, ORAI3, ST3GAL5, ST8SIA4, ST8SIA6, STEAP1  STEAP2, TNFRSF14, TYROBP, XKR6, YME1L1, ZFP14, ABHD6, ACSL4, ADORA3, AMIGO2, ADRA1D, ADRB2  ACER3, AMHR2, AQP10, ALOX5AP, B4GALNT3, BMPR1A  CDH6, CDH7, CALCRL, CAMLG, CACNA2D1, CHST12  CPM, CEACAM1, CLIC6, CLCN6, C1orf162, C16orf54, C6orf25, F8, CSF2RA, CSF2RB, C3AR1,CR1, CNST, CPOX  CYSLTR1, CTNS, CYB561D2, CRLF2, DGAT1, DRD2, EVI2A  EVI2B, ENPP3, ERVFRD-1, EGFR, ERMAP, EPB41, EPOR  EXTL3, FMO4, FLT3LG, FLT3, GABRB2, GABRE, GDPD1  HCST, HAVCR2, HRH4, HILPDA, ITPR1, IGF1R, ITGA2, ITGA4, ITGA6, ITGA9, ITGAL, ITGAM, ITGAV, ITGB4  ITGB6, ICAM3, IFITM2, IL1RAPL1, IL1RL1, IL12RB1, IL12RB2, IL18R1, IL18RAP, IL3RA, IL5RA, IL9R, KLRG1  LILRA2, LAT, LINC00493, LAX1, LPAR5, LPCAT2, LAPTM5  MFSD6L, MR1, HLA-DPA1, HLA-DRB1, HLA-DRB3  HLA-DRB5, MAL2, MAN1A1, MCEMP1, MS4A2, MS4A4E  METTL7A, MAOB, MUC15, MUC16, MCTP1, MCTP2  MYOF, NRROS, NBAS, NETO2, OCLN, OR1L3, OR1L8  OR10H5, OR13A1, OR14K1, OR2AK2, OR2L13, OR2L2  OR2M3, OR2M7, OR5B21, PMP22, PLN, PAG1, PLXNA2  KCNMA1, KCNN1, KCNQ1, PTGER3, PMEPA1, PROS1  PTPRC, PTPRK, PCDHB5, PCDHB6, P2RX1, P2RX4, P2RY1  P2RY14, RNF122, RNF130, RNF144A, RYR2, SELL, SELPLG  SIGLEC14, SIGLEC5, SIGLEC6, SIGLEC7, SIGLEC8, SIGLEC9, SPN, SFXN3, SLAMF1, SMIM3, SCN3A, SCN7A  SCN9A, SLC10A5, SLC17A7, SLC18A2, SLC2A3, SLC22A3, SLC24A3, SLC25A30, SLC27A2, SLC28A3, SLC35F1, SLC39A11, SLC4A4, SLC43A3, SLC45A3, SLC46A3 |
| --- | --- | --- | --- |
| Signal | 5.9E-2 | 7.84 | ATP8B4, CCL1, CCL2, CCL3L3, CCL4L2, CD2, CD200R1  CD22, CD226, CD24, CD300LF, CD300A, CD33  CD48, CD52, CD82, CD84, F2RL3, FCER1A, FCER1G  FCGR2A, FCGR2B, GPR162, GM2A, KIAA0319  KIT, L1CAM, MAS1L, ASAH1, NPC2, ST8SIA4, TRAV8-3  TRGV3, TRGV5, TNFRSF14, TYROBP, AMIGO2  ANGPT2, AMHR2, APOC1, BMP6, BMPR1A, BCAN, CDH6, CDH7, CALCRL, CACNA2D1, CASQ1, CA11, CPM, CEACAM1  CEACAM5, CTSG, CTSS, CTSW, C6orf25, F8, CSF2RA, CSF2RB, CSF2, CR1, CTNS, CRLF2, DPEP2, EVI2A, EVI2B  EMILIN2, ERVFRD-1, ENDOD1, ERP27, EGFR, ERMAP, EPOR  ECM1, FLT3LG, FLT3, FUCA1, GALC, GABRB2, GABRE  GML, GPC3, GDF2, HSPA13, HCST, HAVCR2, HTN1, HSD17B11, HSD17B13, IGKV2-24, INHBB  IGF1R, ITGA2, ITGA4, ITGA6, ITGA9, ITGAL, ITGAM  ITGAV, ITGB4, ITGB6, ICAM3, IFNE, IL1RAPL1, IRAK3  IL1RL1, IL12RB1, IL12RB2, IL18R1, IL18RAP, IL3RA, IL5RA, IL9R, LAMA5, LILRA2, MR1, HLA-DPA1, HLA-DRB1, HLA-DRB3, HLA-DRB5, MANBA, METTL7A, MTFR1L, MUC15, NRROS, NPNT, NRN1, NBEA, NETO2, PTHLH, PTX3, PLA2G2A, PLA2G15, PLAT, PLXNA2, PTGS1, PTGS2  PROS1, PTPRC, PTPRK, PRTN3, PRG2, PCDHB5, PCDHB6, QPRT, RNASE6, RNASET2, RNF130, SELL, SELPLG, SEMA3C, SERPINI1, SIGLEC14, SIGLEC5, SIGLEC6, SIGLEC7, SIGLEC8, SIGLEC9, SPN, SLAMF1, SLC10A5, SLC24A3, SLC46A3, SLC5A12, SLC8A3, SLC9A3, SLCO2B1, SULF2, TNS4, TRANK1, TCN1, TCN2, TGFB1, TMEM145, TMEM154, TPSB2, TPSG1, UTS2, VCAN |
| Integrin mediated signalling pathway | 1.2E-5 | 3.21 | FCER1G, TXK, TYROBP, CEACAM1, C6orf25  FERMT3, ITGA2, ITGA4, ITGA6, ITGA9, ITGAL, ITGAM, ITGAV, ITGB4, ITGB6, LAMA5, LAT, PLEK, VAV1, VAV3 |
| Inflammatory response | 1.4E-3 | 4 | BTK, FCER1A, FCER1G, CSF2, INPP5D, LAT, LCP2, MS4A2, PIK3CG, PIK3R1, PLCG2, PRKCB, VAV1, VAV3 |
| Disulphide bond | 2.2E-4 | 7.84 | HMGCL, CCL1, CCL2, CCL3L3, CCL4L2, CCR2, CD2, CD200R1, CD22, CD226, CD300LF, CD300A, CD33  CD48, CD69, CD84, F2RL3, FASLG, FCER1A, FCER1G  FCGR2A, FCGR2B, GPR34, GPR65, GPR85, GM2A, KIT, KEL  L1CAM, MFNG, GNPTAB, NDST2, NME8, NPC2, RAB27B  ST3GAL5, ST8SIA4, ST8SIA6, TRGV3, TNFRSF14, TYROBP  ADORA3, AMIGO2, ADRB2, ANGPT2, AMHR2, BMP6  BMPR1A, BCAN, CALCRL, CACNA2D1, CPM, CEACAM1  CTSG, CTSS, CTSWF8, CSF2RA, CSF2RB, CSF2, C3AR1  CR1, CYSLTR1, CRLF2, DPEP2, DRD2, ENPP3, EMILIN2  ERVFRD-1, EGFR, ERMAP, EPOR, EXTL3, FLT3LG, FLT3  GALC, GABRB2, GABRE, GML, GDF2, HCST, HAVCR2  HRH4, INHBB, IGF1R, ITGA2, ITGA4, ITGA6, ITGA9, ITGAL, ITGAM, ITGAV, ITGB4, ITGB6, ICAM3, IFNE  IL1RAPL1, IL1RL1, IL12RB1, IL12RB2, L18R1, L18RAP, IL5RA, KLRG1, LAMA5, LILRA2, LPAR5, MR1, HLA-DPA1  HLA-DRB1, HLA-DRB3, HLA-DRB5, MAN1A1, MS4A2  MUC16, NPNT, NETO2, OR1L3, OR1L8, OR10H5, OR13A1  OR14K1, OR2AK2, OR2L13, OR2L2, OR2M3, OR2M7  OR5B21, PTX3, PLA2G2A, PLA2G15, PLAT, PLXNA2, PTGS1, PTGS2, PROS1, PTPN22, PTPRK, PRTN3, PRG2  P2RX1, P2RX4, P2RY1, P2RY14, RNASE6, RNASET2  SELL, SELPLG, SEMA3C, SGK1, SIGLEC14, SIGLEC5  SIGLEC6, SIGLEC7, SIGLEC8, SIGLEC9, SLAMF1, SLC18A2  SLC4A4, SLC7A11, SLCO2B1, SUCNR1, TTN, TCN1, TCN2  TGFB1, TPSB2, TPSG1, UTS2, VCAN |
