# Supplementary material for: Mast Cell Infiltration in Human Brain Metastases Modulates the Microenvironment and Contributes to the Metastatic Potential
Source: Front Oncol. 2017 Jun 2;7:115. doi: 10.3389/fonc.2017.00115 (PMC5454042; doi:10.3389/fonc.2017.00115)
Supplement: Supplementary file 6 [file Image_1.PDF]

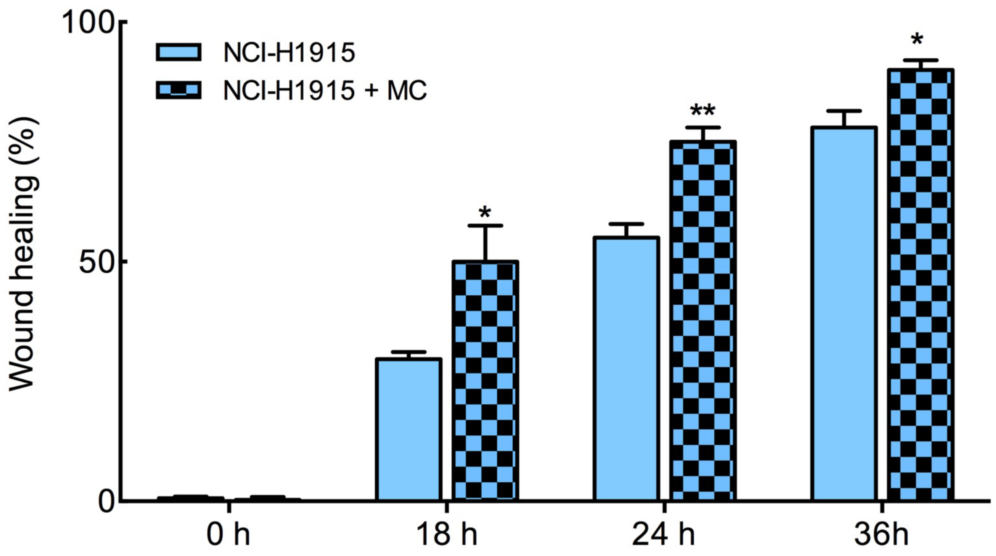

Supplementary Figure S1. MCs can significantly induce migration of BM cells. Migration capacity of the NCI-H1915 cells was assessed by wound healing assay after 18, 24 and 36 hours when grown alone or in co-culture with MCs. The results are shown as percentage of healed wound area. The experiments were performed 3 times in triplicates and mean values + SEM was plotted, * $p < 0.05$, ** $p < 0.01$.
