# Supplementary material for: Mast Cell Infiltration in Human Brain Metastases Modulates the Microenvironment and Contributes to the Metastatic Potential
Source: Front Oncol. 2017 Jun 2;7:115. doi: 10.3389/fonc.2017.00115 (PMC5454042; doi:10.3389/fonc.2017.00115)
Supplement: Supplementary file 7 [file Image_2.PDF]

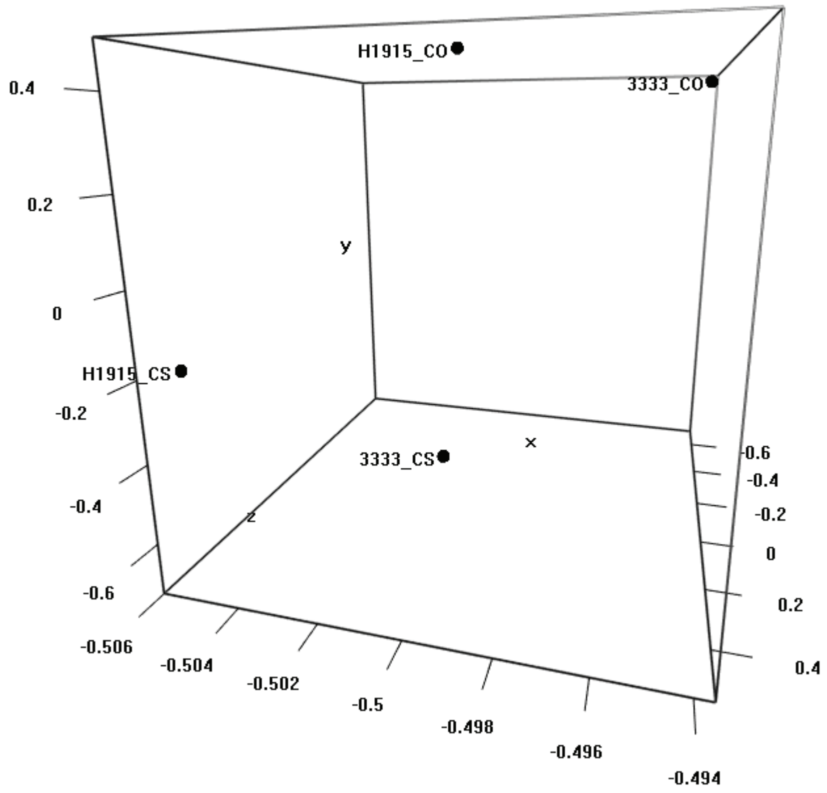

Supplementary Figure S2. A principal component analysis (PCA) was performed to visualize the array data and get an overview of the data set. The controls included in the analysis have been evaluated in Affymetrix Expression Console and the results indicate that the expression profiling has been successful for all samples included in the analysis. H1915_CO = Control NCI-H1915 cells, H1915_CS = LAD2 stimulated NCI-H1915 cells, 3333_CO = Control U3333MET cells, 3333_CS = LAD2 stimulated U3333MET cells.
