# Supplementary material for: Mast Cell Infiltration in Human Brain Metastases Modulates the Microenvironment and Contributes to the Metastatic Potential
Source: Front Oncol. 2017 Jun 2;7:115. doi: 10.3389/fonc.2017.00115 (PMC5454042; doi:10.3389/fonc.2017.00115)
Supplement: Supplementary file 8 [file Image_3.PDF]

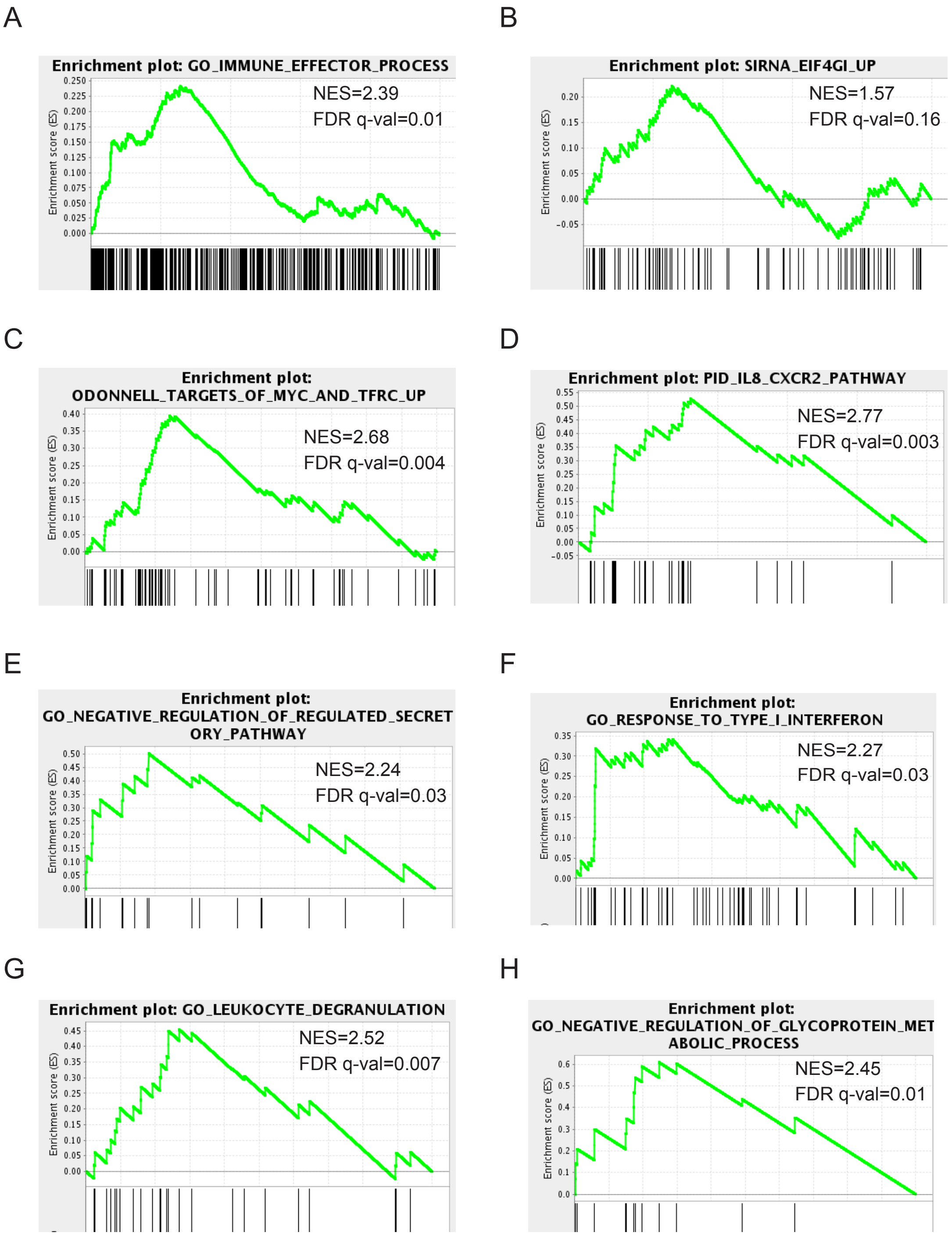


Supplementary Figure S3. Enrichment plots showing profile of the running ES score after GSEA analysis. (A) Enrichment plot: GO_Immune effector process (B) Enrichment plot: SIRNA_ELF4GL_UP (C) Enrichment plot:ODONELL_Targets of MYC and TFRC_UP (D) Enrichment plot: PID_IL-8/ CXCR2 pathway (E) Enrichment plot: GO_ Negative regulation of regulated secretory pathway (F) Enrichment plot: GO_Response to type I interferon (G) Enrichment plot: GO_ Leukocyte degranulation (H) Enrichment plot: GO_Negative regulation of glycoprotein metabolic process.
