# Supplementary figures and images for: Mast Cell Infiltration in Human Brain Metastases Modulates the Microenvironment and Contributes to the Metastatic Potential
Source: Front Oncol. 2017 Jun 2;7:115. doi: 10.3389/fonc.2017.00115 (PMC5454042; doi:10.3389/fonc.2017.00115)

# B

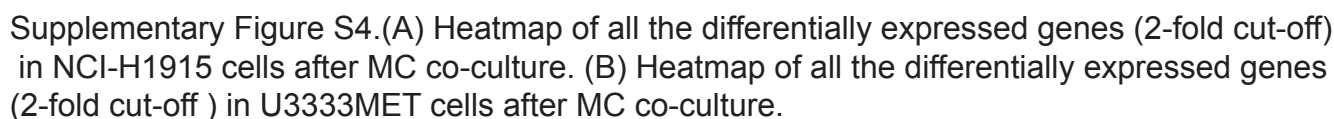

Supplement: Supplementary file 9 [file Image_4.PDF]
