# Supplementary material for: Mast Cell Infiltration in Human Brain Metastases Modulates the Microenvironment and Contributes to the Metastatic Potential
Source: Front Oncol. 2017 Jun 2;7:115. doi: 10.3389/fonc.2017.00115 (PMC5454042; doi:10.3389/fonc.2017.00115)
Supplement: Supplementary file 10 [file Image_5.PDF]

A

Gene Set Name [# Genes (K)]	Description	# Genes in Overlap (k)	k/K	p-value	FDR q-value
RPS14_DN.V1_UP [192]	Genes up-regulated in CD34+ hematopoietic progenitor cells after knockdown of RPS14 [Gene ID=6208] by RNAi.	24		$4.62\ e^{-25}$	$8.73\ e^{-23}$
SNF5_DN.V1_UP [177]	Genes up-regulated in MEF cells (embryonic fibroblasts) with knockout of SNF5 [Gene ID=6598] gene.	14		$2.37\ e^{-12}$	$2.24\ e^{-10}$
P53_DN.V1_DN [192]	Genes down-regulated in NCI-60 panel of cell lines with mutated TP53 [Gene ID=7157].	14		$7.17\ e^{-12}$	$4.52\ e^{-10}$
HOXA9_DN.V1_UP [194]	Genes up-regulated in MOLM-14 cells (AML) with knockdown of HOXA9 [Gene ID=3205] gene by RNAi vs controls.	13		$1.17\ e^{-10}$	$5.54\ e^{-9}$
STK33_SKM_UP [290]	Genes up-regulated in SKM-1 cells (AML) after knockdown of STK33 [Gene ID=65975] by RNAi.	14		$1.66\ e^{-9}$	$6.28\ e^{-8}$
GCNP_SHH_UP_EARLY.V1_DN [169]	Genes down-regulated in granule cell neuron precursors (GCNPs) after stimulation with Shh for 3h.	11		$4.37\ e^{-9}$	$1.38\ e^{-7}$
KRAS.600_UP.V1_UP [287]	Genes up-regulated in four lineages of epithelial cell lines over-expressing an oncogenic form of KRAS [Gene ID=3845] gene.	13		$1.37\ e^{-8}$	$3.69\ e^{-7}$
STK33_UP [293]	Genes up-regulated in NOMO-1 and SKM-1 cells (AML) after knockdown of STK33 [Gene ID=65975] by RNAi.	11		$1.15\ e^{-6}$	$2.73\ e^{-5}$
ATF2_UP.V1_DN [187]	Genes down-regulated in myometrial cells over-expressing ATF2 [Gene ID=1386] gene.	9		$1.45\ e^{-6}$	$3.05\ e^{-5}$
RELA_DN.V1_DN [141]	Genes down-regulated in HEK293 cells (kidney fibroblasts) upon knockdown of RELA [Gene ID=5970] gene by RNAi.	8		$1.65\ e^{-6}$	$3.12\ e^{-5}$

B

Gene Set Name [# Genes (K)]	Description	# Genes in Overlap (k)	k/K	p-value	FDR q-value
GO_SIGNAL_TRANSDUCER_ACTIVITY [1731]	Conveys a signal across a cell to trigger a change in cell function or state. A signal is a physical entity or change in state that is used to transfer information in order to trigger a response.	49		$1.52\ e^{-20}$	$1.41\ e^{-17}$
GO_RECEPTOR_ACTIVITY [1649]	Combining with an extracellular or intracellular messenger to initiate a change in cell activity.	46		$4.71\ e^{-19}$	$2.19\ e^{-16}$
GO_SIGNALING_RECEPTOR_ACTIVITY [1393]	Receiving a signal and transmitting the signal in the cell to initiate a change in cell activity. A signal is a physical entity or change in state that is used to transfer information in order to trigger a response.	39		$2.77\ e^{-16}$	$8.57\ e^{-14}$
GO_MOLECULAR_FUNCTION_REGULATOR [1353]	A molecular function that modulates the activity of a gene product or complex. Examples include enzyme regulators and channel regulators.	38		$6.28\ e^{-16}$	$1.46\ e^{-13}$
GO_GUANYL_NUCLEOTIDE_EXCHANGE_FACTOR_ACTIVITY [303]	Stimulates the exchange of guanyl nucleotides associated with a GTPase. Under normal cellular physiological conditions, the concentration of GTP is higher than that of GDP, favoring the replacement of GDP by GTP in association with the GTPase.	16		$2.96\ e^{-11}$	$5.5\ e^{-9}$
GO_RAS_GUANYL_NUCLEOTIDE_EXCHANGE_FACTOR_ACTIVITY [228]	Stimulates the exchange of guanyl nucleotides associated with a GTPase of the Ras superfamily. Under normal cellular physiological conditions, the concentration of GTP is higher than that of GDP, favoring the replacement of GDP by GTP in association with the GTPase.	14		$7.17\ e^{-11}$	$1.11\ e^{-8}$
GO_NUCLEOSIDE_TRIPHOSPHATASE_REGULATOR_ACTIVITY [329]	Modulates the rate of NTP hydrolysis by a NTPase.	16		$1\ e^{-10}$	$1.33\ e^{-8}$
GO_ENZYME_ACTIVATOR_ACTIVITY [471]	Binds to and increases the activity of an enzyme.	18		$3.32\ e^{-10}$	$3.85\ e^{-8}$
GO_ENZYME_BINDING [1737]	Interacting selectively and non-covalently with any enzyme.	33		$1.73\ e^{-9}$	$1.78\ e^{-7}$
GO_ENZYME_REGULATOR_ACTIVITY [959]	Binds to and modulates the activity of an enzyme.	24		$1.92\ e^{-9}$	$1.78\ e^{-7}$

C

Gene Set Name [# Genes (K)]	Description	# Genes in Overlap (k)	k/K	p-value	FDR q-value
MODULE_45 [583]	Whole blood genes.	56		$2.61\ e^{-51}$	$1.13\ e^{-48}$
MODULE_84 [549]	Immune (humoral) and inflammatory response.	48		$6.14\ e^{-42}$	$1.32\ e^{-39}$
MODULE_15 [358]	Genes in the cancer module 14.	33		$1.07\ e^{-29}$	$1.29\ e^{-27}$
MODULE_44 [327]	Thymus genes.	32		$1.19\ e^{-29}$	$1.29\ e^{-27}$
MODULE_64 [518]	Membranal receptors.	34		$1.13\ e^{-25}$	$9.7\ e^{-24}$
MODULE_75 [399]	Immune response.	29		$2.91\ e^{-23}$	$2.09\ e^{-21}$
MODULE_46 [395]	Genes in the cancer module 46.	26		$6.99\ e^{-20}$	$4.31\ e^{-18}$
MODULE_16 [511]	Genes in the cancer module 15.	28		$3.21\ e^{-19}$	$1.73\ e^{-17}$
MODULE_5 [434]	Lung genes.	26		$7.19\ e^{-19}$	$3.44\ e^{-17}$
MODULE_27 [356]	Genes in the cancer module 27.	21		$2.5\ e^{-15}$	$1.08\ e^{-13}$

Supplementary Figure S5. Computing gene set overlap for the 306 common genes with MSigDB collections. (A) Oncogenic signatures (B) Molecular function (C) Cancer module.
