# Supplementary material for: Mast Cell Infiltration in Human Brain Metastases Modulates the Microenvironment and Contributes to the Metastatic Potential
Source: Front Oncol. 2017 Jun 2;7:115. doi: 10.3389/fonc.2017.00115 (PMC5454042; doi:10.3389/fonc.2017.00115)
Supplement: Supplementary file 11 [file Image_6.PDF]

A

Gene Set Name [# Genes (K)]	Description	# Genes in Overlap (k)	k/K	p-value	FDR q-value
GO_IMMUNE_SYSTEM_PROCESS [1984]	Any process involved in the development or functioning of the immune system, an organismal system for calibrated responses to potential internal or invasive threats.	88		$1.85\ e^{-53}$	$8.61\ e^{-50}$
GO_REGULATION_OF_IMMUNE_SYSTEM_PROCESS [1403]	Any process that modulates the frequency, rate, or extent of an immune system process.	68		$5.14\ e^{-43}$	$1.2\ e^{-39}$
GO_REGULATION_OF_IMMUNE_RESPONSE [858]	Any process that modulates the frequency, rate or extent of the immune response, the immunological reaction of an organism to an immunogenic stimulus.	56		$4.66\ e^{-42}$	$7.23\ e^{-39}$
GO_POSITIVE_REGULATION_OF_IMMUNE_SYSTE STEM_PROCESS [867]	Any process that activates or increases the frequency, rate, or extent of an immune system process.	52		$3.56\ e^{-37}$	$4.14\ e^{-34}$
GO_IMMUNE_RESPONSE [1100]	Any immune system process that functions in the calibrated response of an organism to a potential internal or invasive threat.	53		$3.77\ e^{-33}$	$3.51\ e^{-30}$
GO_POSITIVE_REGULATION_OF_RESPONSE_TO_ TO_STIMULUS [1929]	Any process that activates, maintains or increases the rate of a response to a stimulus. Response to stimulus is a change in state or activity of a cell or an organism (in terms of movement, secretion, enzyme production, gene expression, etc.) as a result of a stimulus.	65		$1.11\ e^{-31}$	$8.61\ e^{-29}$
GO_CELL_ACTIVATION [568]	A change in the morphology or behavior of a cell resulting from exposure to an activating factor such as a cellular or soluble ligand.	40		$2.78\ e^{-31}$	$1.85\ e^{-28}$
GO_REGULATION_OF_CELL_ACTIVATION [484]	Any process that modulates the frequency, rate or extent of cell activation, the change in the morphology or behavior of a cell resulting from exposure to an activating factor such as a cellular or soluble ligand.	37		$2.97\ e^{-30}$	$1.73\ e^{-27}$
GO_REGULATION_OF_CELL_ADHESION [629]	Any process that modulates the frequency, rate or extent of attachment of a cell to another cell or to the extracellular matrix.	40		$1.41\ e^{-29}$	$7.31\ e^{-27}$
GO_BIOLOGICAL_ADHESION [1032]	The attachment of a cell or organism to a substrate, another cell, or other organism. Biological adhesion includes intracellular attachment between membrane regions.	46		$2.57\ e^{-27}$	$1.2\ e^{-24}$

B

Gene Set Name [# Genes (K)]	Description	# Genes in Overlap (k)	k/K	p-value	FDR q-value
RYTTCCTG_V$ETS2_B [1085]	Genes with promoter regions [-2kb,2kb] around transcription start site containing the motif RYTTCCTG which matches annotation for ETS2: v-ets erythroblastosis virus E26 oncogene homolog 2 (avian)	36		$2.34\ e^{-17}$	$1.44\ e^{-14}$
RGAGGAARY_V$PU1_Q6 [502]	Genes with promoter regions [-2kb,2kb] around transcription start site containing the motif RGAGGAARY which matches annotation for SPI1: spleen focus forming virus (SFFV) proviral integration oncogene spi1	23		$2.49\ e^{-14}$	$7.66\ e^{-12}$
V$ETS_Q4 [247]	Genes with promoter regions [-2kb,2kb] around transcription start site containing the motif ANNCACTTCCTG which matches annotation for ETS1: v-ets erythroblastosis virus E26 oncogene homolog 1 (avian)	17		$9.95\ e^{-14}$	$2.04\ e^{-11}$
V$PEA3_Q6 [255]	Genes with promoter regions [-2kb,2kb] around transcription start site containing the motif ACWTCCK which matches annotation for ETV4: ets variant gene 4 (E1A enhancer binding protein, E1AF)	16		$2.21\ e^{-12}$	$3.4\ e^{-10}$
V$PU1_Q6 [234]	Genes with promoter regions [-2kb,2kb] around transcription start site containing the motif WGAGGAAG which matches annotation for SPI1: spleen focus forming virus (SFFV) proviral integration oncogene spi1	14		$1.01\ e^{-10}$	$1.24\ e^{-8}$
RACCACAR_V$AML_Q6 [261]	Genes with promoter regions [-2kb,2kb] around transcription start site containing the motif RACCACAR which matches annotation for RUNX1: runt-related transcription factor 1 (acute myeloid leukemia 1; aml1 oncogene)	14		$4.24\ e^{-10}$	$4.35\ e^{-8}$
V$ELF1_Q6 [244]	Genes with promoter regions [-2kb,2kb] around transcription start site containing the motif RNWMBAGGAART which matches annotation for ELF1: E74-like factor 1 (ets domain transcription factor)	12		$2.01\ e^{-8}$	$1.77\ e^{-6}$
V$AML_Q6 [266]	Genes with promoter regions [-2kb,2kb] around transcription start site containing the motif NNGKNTGTGGTTWNC which matches annotation for RUNX1: runt-related transcription factor 1 (acute myeloid leukemia 1; aml1 oncogene)	12		$5.19\ e^{-8}$	$3.99\ e^{-6}$
V$ETS2_B [274]	Genes with promoter regions [-2kb,2kb] around transcription start site containing the motif KRCAGGAARTRNKT which matches annotation for ETS2: v-ets erythroblastosis virus E26 oncogene homolog 2 (avian)	12		$7.17\ e^{-8}$	$4.9\ e^{-6}$
MGGAAGTG_V$GABP_B [757]	Genes with promoter regions [-2kb,2kb] around transcription start site containing the motif MGGAAGTG which matches annotation for GABPA: GA binding protein transcription factor, alpha subunit 60kDa GABPB2: GA binding protein transcription factor, beta subunit 2	19		$9.44\ e^{-8}$	$5.8\ e^{-6}$

Supplementary Figure S6. Computing gene set overlap for the 306 common genes with MSigDB collections. (A) Biological process (B) Transcription factor targets.
