# Supplementary material for: Mast Cell Infiltration in Human Brain Metastases Modulates the Microenvironment and Contributes to the Metastatic Potential
Source: Front Oncol. 2017 Jun 2;7:115. doi: 10.3389/fonc.2017.00115 (PMC5454042; doi:10.3389/fonc.2017.00115)
Supplement: Supplementary file 12 [file Image_7.PDF]

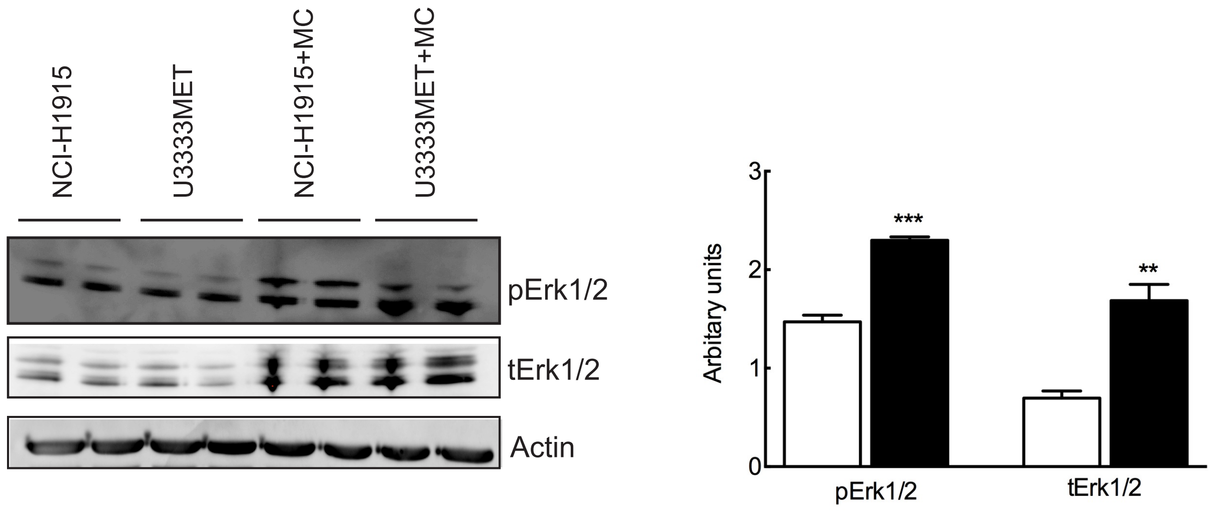

Supplementary Figure S7. MCs association in BM development involves activation of the MAPK (ERK1/2) signaling pathway. Left panel: Representative picture for western blot of phospho Erk1/2 and total Erk1/2 in BM cells (NCI-H1915 and U3333MET cultured alone) and BM cells + MC (NCI-H1915 and U3333MET cells after co-culture with MCs). Right panel: Quantification of pErk1/2 and tErk1/2.
